# Measurement invariance of six language versions of the post-traumatic stress disorder checklist for DSM-5 in civilians after traumatic brain injury

**DOI:** 10.1038/s41598-022-20170-2

**Published:** 2022-10-04

**Authors:** Fabian Bockhop, Marina Zeldovich, Katrin Cunitz, Dominique Van Praag, Marjolein van der Vlegel, Tim Beissbarth, York Hagmayer, Nicole von Steinbuechel, CENTER-TBI participants and investigators, Cecilia Åkerlund, Krisztina Amrein, Nada Andelic, Lasse Andreassen, Audny Anke, Anna Antoni, Gérard Audibert, Philippe Azouvi, Maria Luisa Azzolini, Ronald Bartels, Pál Barzó, Romuald Beauvais, Ronny Beer, Bo-Michael Bellander, Antonio Belli, Habib Benali, Maurizio Berardino, Luigi Beretta, Morten Blaabjerg, Peter Bragge, Alexandra Brazinova, Vibeke Brinck, Joanne Brooker, Camilla Brorsson, Andras Buki, Monika Bullinger, Manuel Cabeleira, Alessio Caccioppola, Emiliana Calappi, Maria Rosa Calvi, Peter Cameron, Guillermo Carbayo Lozano, Marco Carbonara, Simona Cavallo, Giorgio Chevallard, Arturo Chieregato, Giuseppe Citerio, Hans Clusmann, Mark Coburn, Jonathan Coles, Jamie D. Cooper, Marta Correia, Amra Čović, Nicola Curry, Endre Czeiter, Marek Czosnyka, Claire Dahyot-Fizelier, Paul Dark, Helen Dawes, Véronique De Keyser, Vincent Degos, Francesco Della Corte, Hugo den Boogert, Bart Depreitere, Đula Đilvesi, Abhishek Dixit, Emma Donoghue, Jens Dreier, Guy-Loup Dulière, Ari Ercole, Patrick Esser, Erzsébet Ezer, Martin Fabricius, Valery L. Feigin, Kelly Foks, Shirin Frisvold, Alex Furmanov, Pablo Gagliardo, Damien Galanaud, Dashiell Gantner, Guoyi Gao, Pradeep George, Alexandre Ghuysen, Lelde Giga, Ben Glocker, Jagoš Golubovic, Pedro A. Gomez, Johannes Gratz, Benjamin Gravesteijn, Francesca Grossi, Russell L. Gruen, Deepak Gupta, Juanita A. Haagsma, Iain Haitsma, Raimund Helbok, Eirik Helseth, Lindsay Horton, Jilske Huijben, Peter J. Hutchinson, Bram Jacobs, Stefan Jankowski, Mike Jarrett, Ji-yao Jiang, Faye Johnson, Kelly Jones, Mladen Karan, Angelos G. Kolias, Erwin Kompanje, Daniel Kondziella, Evgenios Kornaropoulos, Lars-Owe Koskinen, Noémi Kovács, Ana Kowark, Alfonso Lagares, Linda Lanyon, Steven Laureys, Fiona Lecky, Didier Ledoux, Rolf Lefering, Valerie Legrand, Aurelie Lejeune, Leon Levi, Roger Lightfoot, Hester Lingsma, Andrew I.R. Maas, Ana M. Castaño-León, Marc Maegele, Marek Majdan, Alex Manara, Geoffrey Manley, Costanza Martino, Hugues Maréchal, Julia Mattern, Catherine McMahon, Béla Melegh, David Menon, Tomas Menovsky, Ana Mikolic, Benoit Misset, Visakh Muraleedharan, Lynnette Murray, Ancuta Negru, David Nelson, Virginia Newcombe, Daan Nieboer, József Nyirádi, Otesile Olubukola, Matej Oresic, Fabrizio Ortolano, Aarno Palotie, Paul M. Parizel, Jean-François Payen, Natascha Perera, Vincent Perlbarg, Paolo Persona, Wilco Peul, Anna Piippo-Karjalainen, Matti Pirinen, Dana Pisica, Horia Ples, Suzanne Polinder, Inigo Pomposo, Jussi P. Posti, Louis Puybasset, Andreea Radoi, Arminas Ragauskas, Rahul Raj, Malinka Rambadagalla, Isabel Retel Helmrich, Jonathan Rhodes, Sylvia Richardson, Sophie Richter, Samuli Ripatti, Saulius Rocka, Cecilie Roe, Olav Roise, Jonathan Rosand, Jeffrey V. Rosenfeld, Christina Rosenlund, Guy Rosenthal, Rolf Rossaint, Sandra Rossi, Daniel RueckertMartin Rusnák, Juan Sahuquillo, Oliver Sakowitz, Renan Sanchez-Porras, Janos Sandor, Nadine Schäfer, Silke Schmidt, Herbert Schoechl, Guus Schoonman, Rico Frederik Schou, Elisabeth Schwendenwein, Charlie Sewalt, Ranjit D. Singh, Toril Skandsen, Peter Smielewski, Abayomi Sorinola, Emmanuel Stamatakis, Simon Stanworth, Robert Stevens, William Stewart, Ewout W. Steyerberg, Nino Stocchetti, Nina Sundström, Riikka Takala, Viktória Tamás, Tomas Tamosuitis, Mark Steven Taylor, Braden Te Ao, Olli Tenovuo, Alice Theadom, Matt Thomas, Dick Tibboel, Marjolein Timmers, Christos Tolias, Tony Trapani, Cristina Maria Tudora, Andreas Unterberg, Peter Vajkoczy, Shirley Vallance, Egils Valeinis, Zoltán Vámos, Mathieu van der Jagt, Gregory Van der Steen, Joukje van der Naalt, Jeroen T.J.M. van Dijck, Inge A. M. van Erp, Thomas A. van Essen, Wim Van Hecke, Caroline van Heugten, Dominique Van Praag, Ernest van Veen, Thijs Vande Vyvere, Roel P. J. van Wijk, Alessia Vargiolu, Emmanuel Vega, Kimberley Velt, Jan Verheyden, Paul M. Vespa, Anne Vik, Rimantas Vilcinis, Victor Volovici, Nicole von Steinbüchel, Daphne Voormolen, Petar Vulekovic, Kevin K.W. Wang, Daniel Whitehouse, Eveline Wiegers, Guy Williams, Lindsay Wilson, Stefan Winzeck, Stefan Wolf, Zhihui Yang, Peter Ylén, Alexander Younsi, Frederick A. Zeiler, Veronika Zelinkova, Agate Ziverte, Tommaso Zoerle

**Affiliations:** 1 Institute of Medical Psychology and Medical Sociology, University Medical Center Göttingen, Waldweg 37A, 37073 Göttingen, Germany; 2 Department of Psychology, Antwerp University Hospital and University of Antwerp, Edegem, Belgium; 3 Department of Public Health, Erasmus MC, Rotterdam, The Netherlands; 4 Department of Medical Bioinformatics, University Medical Center, Göttingen, Germany; 5 Georg-Elias-Mueller Institute for Psychology, Georg-August-University, Göttingen, Germany; 6 Department of Neurosurgery, Antwerp University Hospital, Edegem, Belgium; 7 Department of Physiology and Pharmacology, Section of Perioperative Medicine and Intensive Care, Karolinska Institutet, Stockholm, Sweden; 8 János Szentágothai Research Centre, University of Pécs, Pécs, Hungary; 9 Division of Surgery and Clinical Neuroscience, Department of Physical Medicine and Rehabilitation, Oslo University Hospital and University of Oslo, Oslo, Norway; 10 Department of Neurosurgery, University Hospital Northern Norway, Tromso, Norway; 11 Department of Physical Medicine and Rehabilitation, University Hospital Northern Norway, Tromso, Norway; 12 Trauma Surgery, Medical University Vienna, Vienna, Austria; 13 Department of Anesthesiology & Intensive Care, University Hospital Nancy, Nancy, France; 14 Raymond Poincare Hospital, Paris, France; 15 Department of Anesthesiology & Intensive Care, S Raffaele University Hospital, Milan, Italy; 16 Department of Neurosurgery, Radboud University Medical Center, Nijmegen, The Netherlands; 17 Department of Neurosurgery, University of Szeged, Szeged, Hungary; 18 International Projects Management, ARTTIC, Munchen, Germany; 19 Department of Neurology, Neurological Intensive Care Unit, Medical University of Innsbruck, Innsbruck, Austria; 20 Department of Neurosurgery & Anesthesia & Intensive Care Medicine, Karolinska University Hospital, Stockholm, Sweden; 21 NIHR Surgical Reconstruction and Microbiology Research Centre, Birmingham, UK; 22 Anesthesie-Réanimation, Paris, France; 23 Department of Anesthesia & ICU, AOU Città Della Salute E Della Scienza Di Torino - Orthopedic and Trauma Center, Torino, Italy; 24 Department of Neurology, Odense University Hospital, Odense, Denmark; 25 BehaviourWorks Australia, Monash Sustainability Institute, Monash University, Victoria, Australia; 26 Department of Public Health, Faculty of Health Sciences and Social Work, Trnava University, Trnava, Slovakia; 27 Quesgen Systems Inc, Burlingame, CA USA; 28 Australian & New Zealand Intensive Care Research Centre, Department of Epidemiology and Preventive Medicine, School of Public Healthand Preventive Medicine, Monash University, Melbourne, Australia; 29 Department of Surgery and Perioperative Science, Umeå University, Umeå, Sweden; 30 Department of Neurosurgery, Medical School, University of Pécs, Pecs, Hungary; 31 Neurotrauma Research Group, János Szentágothai ResearchCentre, University of Pécs, Pecs, Hungary; 32 Department of Medical Psychology, Universitätsklinikum Hamburg-Eppendorf, Hamburg, Germany; 33 Brain Physics Lab, Division of Neurosurgery, Dept of Clinical Neurosciences, University of Cambridge, Addenbrooke’s Hospital, Cambridge, UK; 34 Neuro ICU, Fondazione IRCCS Cà Granda Ospedale Maggiore Policlinico, Milan, Italy; 35 ANZIC Research Centre, Department of Epidemiology and Preventive Medicine, Monash University, Melbourne, VIC Australia; 36 Department of Neurosurgery, Hospital of Cruces, Bilbao, Spain; 37 NeuroIntensive Care, Niguarda Hospital, Milan, Italy; 38 School of Medicine and Surgery, Università Milano Bicocca, Milano, Italy; 39 NeuroIntensive Care, ASST Di Monza, Monza, Italy; 40 Department of Neurosurgery, Medical Faculty, RWTH Aachen University, Aachen, Germany; 41 Department of Anesthesiology and Intensive Care Medicine, University Hospital Bonn, Bonn, Germany; 42 Department of Anesthesia and Neurointensive Care, Cambridge University Hospital NHS Foundation Trust, Cambridge, UK; 43 School of Public Health & PM, Monash University and The Alfred Hospital, Melbourne, VIC Australia; 44 Radiology/MRI Department, MRC Cognition and Brain Sciences Unit, Cambridge, UK; 45 Oxford University Hospitals NHS Trust, Oxford, UK; 46 Intensive Care Unit, CHU Poitiers, Potiers, France; 47 Critical Care Directorate, University of Manchester NIHR Biomedical Research Centre, Salford Royal Hospital NHS Foundation Trust, Salford, UK; 48 Movement Science Group, Faculty of Health and Life Sciences, Oxford Brookes University, Oxford, UK; 49 Department of Neurosurgery, Antwerp University Hospital and University of Antwerp, Edegem, Belgium; 50 Department of Anesthesia & Intensive Care, Maggiore Della Carità Hospital, Novara, Italy; 51 Department of Neurosurgery, University Hospitals Leuven, Leuven, Belgium; 52 Department of Neurosurgery, Faculty of Medicine, Clinical Centre of Vojvodina, University of Novi Sad, Novi Sad, Serbia; 53 Division of Anaesthesia, University of Cambridge, Addenbrooke’s Hospital, Cambridge, UK; 54 Center for Stroke Research Berlin, , Charité – Universitätsmedizin Berlin, corporate member of Freie Universität Berlin, Humboldt-Universität Zu Berlin, and Berlin Institute of Health, Berlin, Germany; 55 Intensive Care Unit, CHR Citadelle, Liège, Belgium; 56 Department of Anaesthesiology and Intensive Therapy, University of Pécs, Pecs, Hungary; 57 Departments of Neurology, Clinical Neurophysiology and Neuroanesthesiology, Region Hovedstaden Rigshospitalet, Copenhagen, Denmark; 58 National Institute for Stroke and Applied Neurosciences, Faculty of Health and Environmental Studies, Auckland University of Technology, Auckland, New Zealand; 59 Department of Neurology, Erasmus MC, Rotterdam, the Netherlands; 60 Department of Anesthesiology and Intensive Care, University Hospital Northern Norway, Tromso, Norway; 61 Department of Neurosurgery, Hadassah-Hebrew University Medical Center, Jerusalem, Israel; 62 Fundación Instituto Valenciano de Neurorrehabilitación (FIVAN), Valencia, Spain; 63 Department of Neurosurgery, Shanghai Renji Hospital, Shanghai Jiaotong University/School of Medicine, Shanghai, China; 64 Karolinska Institutet, INCF International Neuroinformatics Coordinating Facility, Stockholm, Sweden; 65 Emergency Department, CHU, Liège, Belgium; 66 Neurosurgery Clinic, Pauls Stradins Clinical University Hospital, Riga, Latvia; 67 Department of Computing, Imperial College London, London, UK; 68 Department of Neurosurgery, Hospital Universitario, 12 de Octubre, Madrid, Spain; 69 Department of Anesthesia, Critical Care and Pain Medicine, Medical University of Vienna, Vienna, Austria; 70 College of Health and Medicine, Australian National University, Canberra, Australia; 71 Department of Neurosurgery, Neurosciences Centre and JPN Apex Trauma Centre, Institute of Medical Sciences, New Delhi, 110029 India; 72 Department of Neurosurgery, Erasmus MC, Rotterdam, the Netherlands; 73 Department of Neurosurgery, Oslo University Hospital, Oslo, Norway; 74 Division of Psychology, University of Stirling, Stirling, UK; 75 Division of Neurosurgery, Department of Clinical Neurosciences, Addenbrooke’s Hospital, University of Cambridge, Cambridge, UK; 76 Department of Neurology, University of Groningen, University Medical Center Groningen, Groningen, The Netherlands; 77 Neurointensive Care,, Sheffield Teaching Hospitals NHS Foundation Trust, Sheffield, UK; 78 Salford Royal Hospital NHS Foundation Trust Acute Research Delivery Team, Salford, UK; 79 Department of Intensive Care and Department of Ethics and Philosophy of Medicine, Erasmus Medical Center, Rotterdam, The Netherlands; 80 Department of Clinical Neuroscience, Neurosurgery, Umeå University, Umeå, Sweden; 81 Hungarian Brain Research Program - Grant No, University of Pécs, KTIA_13_NAP-A-II/8, Pecs, Hungary; 82 Department of Anaesthesiology, University Hospital of Aachen, Aachen, Germany; 83 Cyclotron Research Center, University of Liège, Liège, Belgium; 84 Centre for Urgent and Emergency Care Research (CURE), Health Services Research Section, School of Health and Related Research (ScHARR), University of Sheffield, Sheffield, UK; 85 Emergency Department, Salford Royal Hospital, Salford, UK; 86 Institute of Research in Operative Medicine (IFOM), Witten/Herdecke University, Cologne, Germany; 87 VP Global Project Management CNS, ICON, Paris, France; 88 Department of Anesthesiology-Intensive Care, Lille University Hospital, Lille, France; 89 Department of Neurosurgery, Rambam Medical Center, Haifa, Israel; 90 Department of Anesthesiology & Intensive Care, University Hospitals Southhampton NHS Trust, Southhampton, UK; 91 Cologne-Merheim Medical Center (CMMC), Department of Traumatology, Orthopedic Surgery and Sportmedicine, Witten/Herdecke University, Cologne, Germany; 92 Intensive Care Unit, Southmead Hospital, Bristol, UK; 93 Department of Neurological Surgery, University of California, San Francisco, CA USA; 94 Department of Anesthesia & Intensive Care, M. Bufalini Hospital, Cesena, Italy; 95 Department of Neurosurgery, University Hospital Heidelberg, Heidelberg, Germany; 96 Department of Neurosurgery, The Walton Centre, NHS Foundation Trust, Liverpool, UK; 97 Department of Medical Genetics, University of Pécs, Pecs, Hungary; 98 Department of Neurosurgery, Emergency County Hospital Timisoara, Timisoara, Romania; 99 School of Medical Sciences, Örebro University, Örebro, Sweden; 100 Institute for Molecular Medicine Finland, University of Helsinki, Helsinki, Finland; 101 Analytic and Translational Genetics Unit, Department of Medicine; Psychiatric and Neurodevelopmental Genetics Unit, Department of Psychiatry; Department of Neurology, Massachusetts General Hospital, Boston, MA USA; 102 Program in Medical and Population Genetics, The Stanley Center for Psychiatric Research, The Broad Institute of MIT and Harvard, Cambridge, MA USA; 103 Department of Radiology, University of Antwerp, Edegem, Belgium; 104 Department of Anesthesiology & Intensive Care, University Hospital of Grenoble, Grenoble, France; 105 Department of Anesthesia & Intensive Care, Azienda Ospedaliera Università di Padova, Padova, Italy; 106 Deptartment of Neurosurgery, Leiden University Medical Center, Leiden, The Netherlands; 107 Department of Neurosurgery, Medical Center Haaglanden, The Hague, The Netherlands; 108 Department of Neurosurgery, Helsinki University Central Hospital, Helsinki, Finland; 109 Division of Clinical Neurosciences, Department of Neurosurgery and Turku Brain Injury Centre, Turku University Hospital and University of Turku, Turku, Finland; 110 Department of Anesthesiology and Critical Care, Pitié -Salpêtrière Teaching Hospital, Assistance Publique, Hôpitaux de Parisand University Pierre et Marie Curie, Paris, France; 111 Neurotraumatology and Neurosurgery Research Unit (UNINN), Vall d’Hebron Research Institute, Barcelona, Spain; 112 Department of Neurosurgery, Kaunas University of Technology and Vilnius University, Vilnius, Lithuania; 113 Department of Neurosurgery, Rezekne Hospital, Latvia; 114 Department of Anaesthesia, Critical Care & Pain Medicine NHS Lothian & University of Edinburg, Edinburgh, UK; 115 Director, MRC Biostatistics Unit, Cambridge Institute of Public Health, Cambridge, UK; 116 Department of Physical Medicine and Rehabilitation, Oslo University Hospital/University of Oslo, Oslo, Norway; 117 Division of Orthopedics, Oslo University Hospital, Oslo, Norway; 118 Institue of Clinical Medicine, Faculty of Medicine, University of Oslo, Oslo, Norway; 119 Broad Institute, Harvard Medical School, Massachusetts General Hospital, Boston, MA USA; 120 National Trauma Research Institute, The Alfred Hospital, Monash University, Melbourne, VIC Australia; 121 Department of Neurosurgery, Odense University Hospital, Odense, Denmark; 122 International Neurotrauma Research Organisation, Vienna, Austria; 123 Klinik Für Neurochirurgie, Klinikum Ludwigsburg, Ludwigsburg, Germany; 124 Division of Biostatistics and Epidemiology, Department of Preventive Medicine, University of Debrecen, Debrecen, Hungary; 125 Department Health and Prevention, University Greifswald, Greifswald, Germany; 126 Department of Anaesthesiology and Intensive Care, AUVA Trauma Hospital, Salzburg, Austria; 127 Department of Neurology, Elisabeth-TweeSteden Ziekenhuis, Tilburg, The Netherlands; 128 Department of Neuroanesthesia and Neurointensive Care, Odense University Hospital, Odense, Denmark; 129 Department of Neuromedicine and Movement Science, Norwegian University of Science and Technology, NTNU, Trondheim, Norway; 130 Department of Physical Medicine and Rehabilitation, St.Olavs Hospital, Trondheim University Hospital, Trondheim, Norway; 131 Department of Neurosurgery, University of Pécs, Pecs, Hungary; 132 Division of Neuroscience Critical Care, John Hopkins University School of Medicine, Baltimore, USA; 133 Department of Neuropathology, Queen Elizabeth University Hospital and University of Glasgow, Glasgow, UK; 134 Department of Department of Biomedical Data Sciences, Leiden University Medical Center, Leiden, The Netherlands; 135 Department of Pathophysiology and Transplantation, Milan University, and Neuroscience ICU, Fondazione IRCCS Cà Granda OspedaleMaggiore Policlinico, Milano, Italy; 136 Department of Radiation Sciences, Biomedical Engineering, Umeå University, Umeå, Sweden; 137 Perioperative Services, Intensive Care Medicine and Pain Management, Turku University Hospital and University of Turku, Turku, Finland; 138 Department of Neurosurgery, Kaunas University of Health Sciences, Kaunas, Lithuania; 139 Intensive Care and Department of Pediatric Surgery, Erasmus Medical Center, Sophia Children’s Hospital, Rotterdam, The Netherlands; 140 Department of Neurosurgery, Kings College London, London, UK; 141 Neurologie, Neurochirurgie und Psychiatrie, Charité – Universitätsmedizin Berlin, Berlin, Germany; 142 Department of Intensive Care Adults, Erasmus MC– University Medical Center Rotterdam, Rotterdam, The Netherlands; 143 icoMetrix NV, Leuven, Belgium; 144 Director of Neurocritical Care, University of California, Los Angeles, USA; 145 Department of Neurosurgery, St.Olavs Hospital, Trondheim University Hospital, Trondheim, Norway; 146 Department of Emergency Medicine, University of Florida, Gainesville, FL USA; 147 Department of Neurosurgery, Charité – Universitätsmedizin Berlin, corporate member of Freie Universität Berlin, Humboldt-Universitätzu Berlin, and Berlin Institute of Health, Berlin, Germany; 148 VTT Technical Research Centre, Tampere, Finland; 149 Section of Neurosurgery, Department of Surgery, Rady Faculty of Health Sciences, University of Manitoba, Winnipeg, MB Canada

**Keywords:** Brain injuries, Post-traumatic stress disorder, Trauma

## Abstract

Traumatic brain injury (TBI) is frequently associated with neuropsychiatric impairments such as symptoms of post-traumatic stress disorder (PTSD), which can be screened using self-report instruments such as the Post-Traumatic Stress Disorder Checklist for DSM-5 (PCL-5). The current study aims to inspect the factorial validity and cross-linguistic equivalence of the PCL-5 in individuals after TBI with differential severity. Data for six language groups (*n* ≥ 200; Dutch, English, Finnish, Italian, Norwegian, Spanish) were extracted from the CENTER-TBI study database. Factorial validity of PTSD was evaluated using confirmatory factor analyses (CFA), and compared between four concurrent structural models. A multi-group CFA approach was utilized to investigate the measurement invariance (MI) of the PCL-5 across languages. All structural models showed satisfactory goodness-of-fit with small between-model variation. The original DSM-5 model for PTSD provided solid evidence of MI across the language groups. The current study underlines the validity of the clinical DSM-5 conceptualization of PTSD and demonstrates the comparability of PCL-5 symptom scores between language versions in individuals after TBI. Future studies should apply MI methods to other sociodemographic (e.g., age, gender) and injury-related (e.g., TBI severity) characteristics to improve the monitoring and clinical care of individuals suffering from PTSD symptoms after TBI.

## Introduction

Traumatic brain injury (TBI) is characterized by an alteration in brain functions, or other cerebral pathology, resulting from an external force^1^. TBI poses a highly relevant challenge for health care systems worldwide with over 50 million prevalent cases globally^2^ and is associated with substantial societal costs as well as individual burden to patients and caregivers^3,4^. In Europe, the number of TBI-related deaths per year is estimated at about 82,000^5^ with incidental falls and road traffic accidents as the main causes of TBI^6^. Although the vast majority of TBI cases (70–90%) are classified as ‘mild’^7^, TBI is commonly associated with elevated rates of long-term neuropsychiatric and cognitive deficits^8,9^.

Post-traumatic stress disorder (PTSD) is among the most frequently reported psychiatric conditions associated with TBI^10^, mediated by various biological and psychological mechanisms^11^. According to the latest version of the Diagnostic and Statistical Manual of Mental Disorders (DSM-5)^12^ PTSD manifests in symptoms of intrusion and hyperarousal among others that emerge after the exposure to actual or threatened death, serious injury, or sexual violence. PTSD prevalence rates range between 0.38% and 6.67% in general populations across Europe related to various trauma causes^13^. Importantly, a recent review by Conroy and colleagues (2020)^14^ noted that while affective disturbances in general are common comorbidities also in other neuropsychiatric diseases (i.e., stroke, Parkinson’s disease, multiple sclerosis), the relationship between PTSD and TBI is particularly distinct since both conditions are likely to emerge from a shared traumatic experience. Indeed, an elevated prevalence of comorbid PTSD (15.6%) can be observed in subjects after TBI, constituting a 73% higher risk of manifestation compared with individuals who exclusively sustained extracranial bodily injuries^15^. However, the emergence of PTSD may pass unnoticed in individuals after TBI due to the overlap in etiology, neuropsychiatric symptoms (e.g., headaches, hypersensitivity, sleep disturbances, impulsivity), and pathophysiological mechanisms between the two conditions^16^. As the treatment of PTSD is associated with major costs and burdens^17,18^, the implementation of valid instruments to evaluate PTSD symptoms is greatly important in order to ensure the appropriate therapy and thereby to substantially reduce the financial and social strain in the field of TBI.

The Post-Traumatic Stress Disorder Checklist (PCL)^19^ is a self-report screening tool for PTSD symptoms that can assist in identifying subjects in need of psychiatric treatment. Its most recent version (PCL-5)^20^ was updated based on the revised DSM-5 diagnostic criteria for PTSD, which propose the underlying disease dimensions following a traumatic experience (criterion A) to be intrusion (criterion B), avoidance (criterion C), negative alterations in cognition and mood (criterion D), and alterations in arousal and reactivity (criterion E). The PCL-5 assesses common symptoms contained in criteria B to E. However, the DSM-5 conceptualization of PTSD has been repeatedly challenged, as concurrent latent dimensions of psychopathology have been put forward by a broad range of factor analytic studies using the PCL-5^21^. To date the debate about the characteristic factor structure of PTSD remains ongoing. In order to ensure valid psychopathological assessments, a thorough investigation of the factorial validity of PTSD measured using the PCL-5 is crucial.

Several language versions of the PCL-5 have been validated and have demonstrated good to excellent psychometric properties^22–30^. Recent work additionally demonstrated the validity of multiple PCL-5 translations in individuals after TBI and reported, for instance, a moderate to strong negative relationship of PTSD symptomatology with subjects’ functional recovery following TBI^31^. However, while instrument translation implies the equivalent assessment of the latent construct (i.e., PTSD) between language versions, empirical evidence for this assumption is required. The concept of measurement invariance (MI)^32^ describes the condition that equal item scores between subjects or groups should convey equal information, so that a lack of MI would result in a biased or misleading interpretation of individual symptomatology^33^. Thus, analyses of MI allow conclusions to be drawn as to whether group differences in mean scores are attributable to a ‘true’ variation in latent symptomatology rather than measurement error or bias. Prior research has demonstrated a lack of comprehensive MI in PTSD symptom scores assessed using a previous version of the PCL (i.e., PCL-C) when comparing military personnel with and without recent deployment^34^. This finding suggested that differences in symptom scores were extensively impacted by variables unrelated to the underlying latent PTSD psychopathology, prompting a further revision of the instrument. With regard to the PCL-5, initial evidence showed fundamental MI of the English and French versions in healthy individuals^35^. However, investigations of MI across multiple language versions of the PCL-5 in populations after TBI have not yet been conducted. Besides cross-linguistic comparisons, studies may utilize MI analyses to further enhance the understanding of PTSD symptomatology within or across the general population and specific clinical samples. First evidence in favor of MI of the PCL-5 in individuals who had experienced a single trauma or multiple trauma types was found^36^, while no evidence for MI in PTSD symptoms scores between trauma-exposed college students and military veterans was observed^37^. These results represent important steps towards the validation of the PCL-5 and its comparability in different populations but evidence across more clinical features is called for. The required data basis for bridging this gap is provided by large-scale international multicenter studies that assess a variety of psychopathological outcome parameters across a wide array of subject characteristics and language groups^38,39^.

The main aims of the current study were to understand the latent factor structure of PTSD in individuals after TBI and to investigate the equivalence of symptom assessments across multiple language versions of the PCL-5 applying MI procedures. Evidence in favor of MI would suggest that the PCL-5 can be used to assess one and the same latent construct of PTSD across all tested languages, allowing for data aggregation and direct comparisons of the PTSD symptomatology after TBI. Finally, MI analyses were conducted in individuals with different levels of recovery and severity of TBI in an effort to strengthen the applicability of the PCL-5 in TBI populations.

## Results

Sociodemographic and injury-related data are presented in Table 1. The total sample comprised 1776 individuals within six language subgroups (Dutch: n = 586, English: n = 213, Finnish: n = 212, Italian: n = 261, Norwegian: n = 248, Spanish: n = 256). Notable variations were observed in the descriptive characteristics. Most prominently, the proportion of participants with a previous history of psychological problems in the English subsample (22.07%) was distinctly above the total average (12.27%). Moreover, reference of the PCL-5 to the TBI experience was reported only by a minority of Dutch-speaking individuals (39.08%) in contrast to the remaining language groups (72.30–84.77%). Pronounced PTSD symptomatology (i.e., PCL-5 ≥ 31) was present in 10.7% of all participants, with the lowest proportion in the Finnish (7.08%) and the highest rate in the Italian (17.24%) subsamples. Statistical analyses by means of ANOVA and Kruskal–Wallis tests showed that Dutch-speaking subjects were significantly older compared with most other language groups, Finnish individuals presented more favorable recovery and fewer extracranial injuries, and the Italian subsample suffered from more severe TBI and PTSD symptoms. However, the overall effect sizes of the observed differences were small (ds: 0.19–0.36) (Table A1 in Appendix). For an overview of the sociodemographic and injury-related characteristics in individuals after ‘ultra-mild’ or more severe TBI, see Table D1 in the Appendix.

**Table 1:** Demographic and clinical characteristics of the total sample and the language subsamples.

	Total	Dutch	English	Finnish	Italian	Norwegian	Spanish
**No. of cases**							
N (% of total)	1776 (100.00)	586 (33.00)	213 (11.99)	212 (11.94)	261 (14.70)	248 (13.96)	256 (14.41)
**Age**							
M (SD)	49.44 (19.43)	52.97 (19.04)	47.90 (16.99)	47.81 (19.57)	49.67 (20.69)	45.97 (19.68)	47.12 (19.42)
Mdn (min; max)	51 (16; 95)	57 (16; 95)	51 (16; 85)	50 (16; 89)	53 (16; 93)	48 (16; 89)	44 (16; 95)
**Gender**							
Female	621 (34.97)	226 (38.57)	69 (32.39)	86 (40.57)	83 (31.80)	80 (32.26)	77 (30.08)
Male	1155 (65.03)	360 (61.43)	144 (67.61)	126 (59.43)	178 (68.20)	168 (67.74)	179 (69.92)
**Living situation**							
Alone	367 (20.66)	138 (23.55)	45 (21.13)	61 (28.77)	38 (14.56)	48 (19.35)	37 (14.45)
Not alone	1409 (79.34)	448 (76.45)	168 (78.87)	151 (71.23)	223 (85.44)	200 (80.65)	219 (85.55)
**Education**							
None/primary	236 (13.29)	40 (6.83)	2 (0.94)	23 (10.85)	63 (24.14)	25 (10.08)	83 (32.42)
Secondary	515 (29.00)	123 (20.99)	57 (26.76)	70 (33.02)	98 (37.55)	61 (24.60)	106 (41.41)
Post-secondary	843 (47.47)	357 (60.92)	134 (62.91)	73 (34.43)	63 (24.14)	152 (61.29)	64 (25.00)
NA	182 (10.24)	66 (11.26)	20 (9.39)	46 (21.70)	37 (14.17)	10 (4.03)	3 (1.17)
**Pre-TBI employment**							
Full-time	743 (41.84)	187 (31.91)	112 (52.58)	89 (41.98)	102 (39.08)	122 (49.19)	131 (51.17)
Part-time	210 (11.82)	88 (15.02)	22 (10.33)	6 (2.83)	36 (13.79)	32 (12.90)	26 (10.16)
In training	168 (9.46)	50 (8.53)	11 (5.17)	30 (14.15)	27 (10.34)	34 (13.71)	16 (6.25)
Unemployed	129 (7.26)	42 (7.17)	18 (8.45)	14 (6.60)	16 (6.13)	16 (6.45)	23 (8.98)
Retired	411 (23.14)	174 (29.69)	36 (16.90)	51 (24.06)	56 (21.46)	39 (15.73)	55 (21.49)
NA	115 (6.48)	45 (7.68)	14 (6.57)	22 (10.38)	24 (9.20)	5 (2.02)	5 (1.95)
**Pre-TBI psychiatric history**							
Yes	218 (12.28)	57 (9.73)	47 (22.06)	30 (14.15)	23 (8.81)	31 (12.50)	30 (11.72)
No	1545 (86.99)	526 (89.76)	161 (75.59)	182 (85.85)	238 (91.19)	213 (85.89)	225 (87.89)
NA	13 (0.73)	3 (0.51)	5 (2.35)	0 (0.00)	0 (0.00)	4 (1.61)	1 (0.39)
**TBI cause**							
Incidental fall	773 (43.52)	283 (48.30)	80 (37.56)	99 (46.70)	92 (35.25)	102 (41.13)	117 (45.70)
RTA	745 (41.95)	232 (39.59)	101 (47.42)	68 (32.08)	132 (50.58)	103 (41.53)	109 (42.58)
Other	227 (12.78)	66 (11.26)	29 (13.61)	37 (17.45)	30 (11.49)	40 (16.13)	25 (9.77)
NA	31 (1.75)	5 (0.85)	3 (1.41)	8 (3.77)	7 (2.68)	3 (1.21)	5 (1.95)
**Clinical care pathways**							
ER	409 (23.03)	102 (17.40)	56 (26.29)	51 (24.06)	67 (25.67)	61 (24.60)	72 (28.13)
ADM	692 (38.96)	305 (52.05)	68 (31.93)	99 (46.70)	56 (21.46)	110 (44.35)	54 (21.09)
ICU	675 (38.01)	179 (30.55)	89 (41.78)	62 (29.24)	138 (52.87)	77 (31.05)	130 (50.78)
**Loss of consciousness**							
Yes	1044 (58.78)	348 (59.38)	144 (67.60)	138 (65.09)	106 (40.61)	169 (68.15)	139 (54.30)
No	567 (31.93)	196 (33.45)	43 (20.19)	65 (30.66)	127 (48.66)	38 (15.32)	98 (38.28)
NA	165 (9.29)	42 (7.17)	26 (12.21)	9 (4.25)	28 (10.73)	41 (16.53)	19 (7.42)
**TBI severity**							
Uncomplicated mild	614 (34.57)	264 (45.05)	63 (29.58)	61 (28.77)	46 (17.62)	104 (41.94)	76 (29.69)
Complicated mild	536 (30.18)	193 (32.93)	54 (25.35)	57 (26.89)	54 (20.69)	81 (32.66)	97 (37.89)
Moderate	127 (7.15)	41 (7.00)	11 (5.17)	16 (7.55)	31 (11.88)	16 (6.45)	12 (4.69)
Severe	262 (14.75)	60 (10.24)	53 (24.88)	15 (7.07)	57 (21.84)	24 (9.68)	53 (20.70)
NA	237 (13.35)	28 (4.78)	32 (15.02)	63 (29.72)	73 (27.97)	23 (9.27)	18 (7.03)
**GCS at baseline**							
M (SD)	12.94 (3.69)	13.46 (3.05)	11.97 (4.52)	13.71 (2.72)	12.04 (4.08)	13.48 (3.03)	12.35 (4.53)
Mdn (min; max)	15 (3; 15)	15 (3; 15)	15 (3; 15)	15 (3; 15)	14 (3; 15)	15 (3; 15)	15 (3; 15)
**Recovery at 6 months (GOSE)**							
Good recovery	1159 (65.26)	398 (67.92)	114 (53.52)	158 (74.53)	156 (59.77)	157 (63.31)	176 (68.75)
Moderate disability	457 (25.73)	145 (24.74)	69 (32.39)	39 (18.40)	67 (25.67)	83 (33.47)	54 (21.09)
Severe disability	159 (8.95)	43 (7.34)	29 (13.62)	15 (7.07)	38 (14.56)	8 (3.22)	26 (10.16)
NA	1 (0.06)	0 (0.00)	1 (0.47)	0 (0.00)	0 (0.00)	0 (0.00)	0 (0.00)
E**xtracranial injury severity score (ISS)**							
M (SD)	18.16 (14.76)	16.79 (12.04)	21.06 (17.46)	13.17 (9.92)	22.33 (17.82)	17.46 (14.56)	19.44 (16.38)
Mdn (min; max)	13 (1; 75)	13 (1; 75)	18 (1; 75)	9 (1; 50)	18 (1; 75)	13 (1; 75)	16 (1; 75)
**PCL-5 referring to TBI event**							
Yes	1168 (65.77)	229 (39.08)	154 (72.30)	164 (77.36)	219 (83.91)	185 (74.60)	217 (84.77)
No	549 (30.91)	333 (56.83)	50 (23.47)	47 (22.17)	40 (15.32)	41 (16.53)	38 (14.84)
NA	59 (3.32)	24 (4.09)	9 (4.23)	1 (0.47)	2 (0.77)	22 (8.87)	1 (0.39)
**PTSD symptoms (PCL-5)**							
M (SD)	12.12 (13.74)	10.76 (12.91)	13.62 (15.08)	10.22 (11.32)	15.17 (15.10)	10.38 (12.24)	14.16 (15.28)
Mdn (min; max)	7 (0; 72)	6 (0; 72)	8 (0; 71)	6 (0; 55)	10 (0; 65)	7 (0; 62)	9 (0; 68)
**Provisional PTSD (PCL-5 ≥ 31)**							
Yes	190 (10.70)	48 (8.19)	28 (13.15)	15 (7.08)	45 (17.24)	19 (7.66)	35 (13.67)
No	1586 (89.30)	538 (91.81)	185 (86.85)	197 (92.92)	216 (82.76)	229 (92.34)	221 (86.33)

For continuous variables and total scores, mean (M) with standard deviation (SD) and median (Mdn) with range (min; max) are reported; *ADM* admission to ward, *ER* emergency room, *GCS* Glasgow coma scale, *GOSE* Glasgow outcome scale extended, *ICU* intensive care unit, *ISS* injury severity score, *NA* not available, *PCL-5* total score on post-traumatic stress disorder checklist for DSM-5, *PTSD* post-traumatic stress disorder, *RTA* road traffic accident, *TBI* traumatic brain injury.

### Structural Validity

In all four candidate models the majority of items had high loadings (βs ≥ 0.80) on the respective proposed factors. No loadings below the cutoff (β < 0.50) were observed (Table 2). Goodness-of-fit parameters were satisfactory for all tested models (Table 3). The variation in the goodness-of-fit indices showed a slightly better fit for the concurrent models compared with the original DSM-5 model. However, overall differences in model fit were small. Since all candidate models showed a similarly satisfactory fit, subsequent MI analyses were conservatively based on the theory-driven DSM-5 conceptualization of PTSD.

**Table 2:** Standardized factor loadings (β) of PCL-5 items for candidate structure models.

PCL-5 Item	DSM-5 model	Dysphoria model	Anhedonia model	Hybrid model
(B1) Memories	.861	.861	.861	.861
(B2) Dreams	.863	.863	.863	.863
(B3) Flashbacks	.895	.895	.895	.895
(B4) Cued distress	.874	.874	.874	.874
(B5) Cued physical reactions	.896	.896	.896	.896
(C1) Avoiding internal reminders	.915	.915	.915	.915
(C2) Avoiding external reminders	.896	.896	.896	.896
(D1) Dissociative amnesia	.524	.519	.534	.534
(D2) Negative beliefs	.820	.814	.840	.840
(D3) Blame	.693	.688	.705	.704
(D4) Negative feelings	.891	.886	.909	.910
(D5) Loss of interest	.808	.801	.850	.850
(D6) Detachment or estrangement	.867	.860	.907	.907
(D7) Numbing	.850	.844	.891	.891
(E1) Irritability or aggressive behavior	.780	.762	.791	.831
(E2) Reckless behavior	.664	.655	.674	.702
(E3) Hypervigilance	.720	.781	.781	.781
(E4) Startle	.845	.931	.931	.931
(E5) Concentration	.781	.767	.795	.813
(E6) Sleep	.694	.685	.704	.718

*DSM-5* diagnostic and statistical manual of mental disorders 5th edition, *PCL-5* post-traumatic stress disorder checklist for DSM-5.

**Table 3:** CFA results for PTSD structure models across total sample (N = 1776).

Model	No. factors	χ^2^	*df*	*p*	CFI	TLI	SRMR	RMSEA	RMSEA 90% CI	**Δ**CFI	**Δ**TLI	**Δ**SRMR	**Δ**RMSEA	**Δ**χ^2^	**Δ**df	**Δ***p*
DSM-5	4	1148.18	166	< .001	0.993	0.992	0.051	0.058	[0.055; 0.061]	–	–	–	–	–	–	–
Dysphoria	4	818.55	166	< .001	0.995	0.995	0.043	0.047	[0.044; 0.051]	0.002	0.003	–0.008	–0.011	–	–	–
Anhedonia	6	855.49	164	< .001	0.995	0.994	0.045	0.049	[0.046; 0.052]	0.002	0.002	–0.006	–0.009	130.56	2	< .001
Hybrid	7	863.09	163	< .001	0.995	0.994	0.045	0.049	[0.046; 0.053]	0.002	0.002	–0.006	–0.009	187.95	3	< .001

The DSM-5 model served as a reference. Scaled chi-square difference tests were computed between the DSM-5 model and nested models. *CFA* confirmatory factor analysis, *CFI* comparative fit index, ΔCFI, difference in CFI; *CI* confidence interval, *df* degrees of freedom, Δdf, difference in df; *No*. number of, *p* statistical significance of χ^2^, *PTSD* post-traumatic stress disorder, Δp statistical significance of Δχ^2^, *RMSEA* root mean square of approximation, ΔRMSEA, difference in RMSEA; *SRMR* standard root mean square residual, ΔSRMR, difference in SRMR; *TLI* Tucker-Lewis index, ΔTLI, difference in TLI; χ^2^, overall scaled chi-square statistic; Δχ^2^, scaled chi-square difference statistic.

### Measurement invariance

Preparatory data inspection revealed that there were no subjects who used the response category 4 (‘extremely impaired’) with regard to a small number of PCL-5 items in the Finnish (i.e., Flashbacks, Reckless behavior, Cued physical reactions) and Norwegian (i.e., Dreams) subsamples. In keeping with the requirements of the MI approach the response categories 3 (‘quite a bit impaired’) and 4 (‘extremely impaired’) were collapsed for these items in all subsamples. Model fit statistics for the main MI analyses are presented in Table 4. Goodness-of-fit was excellent for all MI models. The variation in the descriptive fit indices was below the respective cutoff values and likelihood ratio tests suggested no significant fit differences between the MI models. Equal fit of all MI models was inferred, thus providing evidence for the structural equivalence of PTSD assessment across the total sample.

**Table 4:** Multi-group CFA results across language groups in total sample (N = 1776).

Model	χ^2^	*df*	*p*	CFI	TLI	SRMR	RMSEA	RMSEA 90% CI	**Δ**CFI	**Δ**TLI	**Δ**SRMR	**Δ**RMSEA	**Δ**χ^2^	**Δ**df	**Δ***p*
Configural	1525.79	984	< .001	0.997	0.996	0.06	0.043	[0.039; 0.048]	–	–	–	–	–	–	–
Thresholds	1623.57	1164	< .001	0.997	0.997	0.06	0.037	[0.032; 0.041]	0.000	0.001	0.000	− 0.006	113.06	180	> .99
Loadings	1696.17	1244	< .001	0.997	0.997	0.06	0.035	[0.031; 0.039]	0.000	0.000	0.000	− 0.002	35.73	80	> .99

Results are based on the original DSM-5 structure of PTSD^12^. When the use of the response range was limited, item categories were collapsed. MI models are increasingly restricted and nested. The previous model always serves as a reference. *CFA* confirmatory factor analysis, *CFI* comparative fit index, ΔCFI, difference in CFI; *CI* confidence interval, *df* degrees of freedom; Δdf, difference in df; *p* statistical significance of χ^2^, Δp, statistical significance of Δχ^2^; *RMSEA* root mean square of approximation; ΔRMSEA, difference in RMSEA; *SRMR* standard root mean square residual; ΔSRMR, difference in SRMR; *TLI* Tucker-Lewis index; ΔTLI, difference in TLI; χ^2^, overall scaled chi-square statistic; Δχ^2^, scaled chi-square difference statistic.

See Table C1 in the Appendix for the results of the complimentary analyses which retained the original response categories across all PCL-5 items in a subset of the total sample (N = 1316), excluding the Finnish and Norwegian subsamples due to their limited use of the response category 4 (‘extremely impaired’) in a few items. Goodness-of-fit was satisfactory for all MI models, the differences between the descriptive fit indices were minimal, and the likelihood ratio tests indicated no significant fit difference. Therefore, complimentary analysis using the original response format across all items underlined the main findings of equivalent PTSD assessment across the tested PCL-5 language versions in civilians after TBI.

With regard to comparisons of ‘ultra-mild’ with more severe TBI cases, data inspection revealed that in the ‘ultra-mild’ group no individuals indicated the response category 4 (‘extremely impaired’) in three items (i.e., Dreams, Irritability or aggressive behavior, Startle). Consequently, the response categories 3 (‘quite a bit impaired’) and 4 (‘extremely impaired’) were collapsed for these items across the ‘ultra-mild’ and more severely impaired individuals. The results of the subsequent MI analyses are presented in Table 5. Goodness-of-fit was excellent for all MI models, minimal differences between the descriptive fit parameters were observed, and the results of the likelihood ratio tests indicated no significant differences in model fit between MI models. Therefore, an equal fit across all MI models was concluded and evidence for the structurally equivalent assessment of PTSD symptoms using the PCL-5 between completely recovered individuals and those who sustained a more severe TBI was obtained.

**Table 5:** Multi-group CFA results for the comparisons of individuals after ‘ultra-mild’ TBI and more severe cases^51^ (N = 1776).

Model	χ^2^	*df*	*p*	CFI	TLI	SRMR	RMSEA	RMSEA 90% CI	**Δ**CFI	**Δ**TLI	**Δ**SRMR	**Δ**RMSEA	**Δ**χ^2^	**Δ**df	**Δ***p*
Configural	929.97	328	< .001	0.996	0.995	0.047	0.046	[0.042; 0.049]	–	–	–	–	–	–	–
Thresholds	942.91	365	< .001	0.996	0.996	0.047	0.042	[0.039; 0.046]	0.000	0.001	0.000	− 0.004	12.33	37	> .99
Loadings	952.84	381	< .001	0.996	0.996	0.047	0.041	[0.038; 0.045]	0.000	0.000	0.000	− 0.001	3.47	16	> .99

Results are based on the original DSM-5 structure of PTSD^12^. When the use of the response range was limited, item categories were collapsed. MI models are increasingly restricted and nested. The previous model always serves as a reference. *CFA* confirmatory factor analysis; *CFI* comparative fit index; ΔCFI, difference in CFI; *CI* confidence interval, *df* degrees of freedom; Δdf, difference in df; *p* statistical significance of χ^2^; Δp, statistical significance of Δχ2; *RMSEA* root mean square of approximation; ΔRMSEA, difference in RMSEA; *SRMR* standard root mean square residual; ΔSRMR, difference in SRMR; *TLI* Tucker-Lewis index; ΔTLI, difference in TLI; χ^2^, overall scaled chi-square statistic; Δχ^2^, scaled chi-square difference statistic.

## Discussion

The current study aimed to examine the latent factorial structure and cross-linguistic invariance of the PCL-5 as an assessment tool for PTSD symptomatology using data collected in the CENTER-TBI study. Although validation has been available for several language versions of the PCL-5^22,31^, this is the first study to evaluate whether measurements of PTSD symptoms were equivalent in six language groups (i.e., Dutch, English, Finnish, Italian, Norwegian, Spanish) of civilians after TBI. PTSD symptomatology was prevalent in all language subsamples with the proportions of highly affected individuals ranging between 7.08 and 17.24%, which is in line with previous reports^15^. Subsequent structural analyses resulted overall in a satisfactory fit for four structural models of PTSD, including the clinical DSM-5 conceptualization. Applying MI procedures conservatively based on the theory-driven DSM-5 model provided solid evidence for equivalent PCL-5 assessments. Therefore, symptom scores both across the tested language versions as well as between individuals after ‘ultra-mild’ or more severe TBI can be considered comparable. The presented evidence points towards the applicability of the PCL-5 in these populations.

In order to reduce the risk of substantial sampling bias when analyzing the generalizability of PTSD measurement across multiple language versions homogeneity of sociodemographic and injury-related variables across subsamples was desirable. The results of the descriptive analyses revealed sufficiently low variability in the current study. Although the injury characteristics differed significantly across language subsamples, the effect sizes were small and the risk of statistical artifacts was elevated by the relatively large subgroup sample size required for MI analyses^40^. Interestingly, individuals in the Dutch-speaking subsample were relatively old, had mostly experienced TBI resulting from incidental falls, often referred their answers in the PCL-5 to traumatic events other than the TBI experience, and showed rather mild symptoms of TBI and PTSD. Based on this observation, the relationship between functional outcomes and descriptive statistics including type, count, and timepoint of trauma needs further investigation. Previous studies have demonstrated the generalizability of PCL-5 assessments across descriptive strata such as gender^41^ or single- and multi-trauma types^36^. Future research should investigate equivalence across additional sociodemographic factors (e.g., age, education)^42,43^, injury-related characteristics (e.g., TBI severity, injury cause, healthy populations and non-TBI patients), and physical comorbidities (e.g., diabetes, cancer)^44^ in order to ensure a conclusive interpretation of symptom scores across subjects with diverse traits in clinical settings.

The current study reproduced previous findings on the structural validity of the PCL-5 in the Dutch subsample in CENTER-TBI^45^, extended the conclusions to five additional language subsamples and found satisfactory goodness-of-fit for the original DSM-5 model as well as concurrent models. As previously shown^23,27,46,47^, the concurrent models exhibited a better fit compared with the DSM-5 model. However, all concurrent models introduced structural factors that comprised fewer than three questionnaire items leading to reduced statistical robustness^48^. The higher number of latent factors in both the Anhedonia and the Hybrid models additionally led to increased model complexity. Thus, further statistical analyses were based conservatively on the theory-driven DSM-5 model which provided robust results that offer strong practical utility. Nonetheless, examinations of latent symptom dimensions add to the understanding of pathological factors central to PTSD and should be studied further to improve therapeutic treatment.

The current study exhibited a number of strengths. Firstly, results were based on high-quality multicenter data that representatively encompassed the complete TBI severity spectrum^7^. Due to this, it was possible to draw reliable conclusions for individuals after TBI. Furthermore, potential sources of bias in the descriptive characteristics across language subsamples were minimal and the factorial structure of the PCL-5 was verified. Finally, this is the first study to date that provides evidence for the comparability of PCL-5 scores across six language versions by applying robust statistical methods to test for MI. Therefore, the reported results uniquely validate comorbid PTSD assessments in the field of TBI.

The present investigation was limited by the inherent overlap of neuropsychiatric symptoms resulting from TBI experience and PTSD symptomatology, thereby posing a confounding effect in PCL-5 assessments^16^. However, while the extent to which scale scores represented expressions of PTSD as opposed to TBI symptomatology remains unclear, assessment of individuals after TBI increased the variance in the PCL-5 scores and prevented floor effects. Interestingly, we observed a lack of extreme impairment with regard to certain PTSD symptoms (i.e., Dreams, Flashbacks, Reckless behavior, Cued physical reactions) in the Finnish and Norwegian subsamples. Although overall differences in TBI severity were small across all language groups, the majority of Finnish and Norwegian individuals suffered from relatively mild TBI. Hence, the manifestation of these particular symptoms as PTSD-specific in contrast to injury-related in populations after TBI should be studied more extensively. Nonetheless, comparability of PCL-5 scores was established by adapting the response categories in the respective items and remained unchanged after the Finnish and Norwegian subsamples had been excluded. Moreover, the MI approach applied in the current study produced reasonable and durable results for the given dataset. Nonetheless, the application of alternative procedures for multi-group equivalence testing in differential data structures should be examined as well, for instance based on Item Response Theory^49^, Exploratory Structural Equation Modelling^50^, or Bifactor Models^21^. Moreover, we employed a previously proposed approach to identify a subset of ‘ultra-mild’ TBI cases^51^ which served as proxies for healthy individuals in the current analyses. However, since subjects in the ‘ultra-mild’ group were still TBI-affected to a certain degree, further investigations based on suitable datasets will be necessary to allow for robust conclusions on the comparability of the PCL-5 between general population samples and individuals after TBI. Finally, since the vast majority of TBI cases are classified as mild and may receive differential treatment of PTSD symptoms among other psychosocial disturbances depending on the inclusion in a specific clinical care pathway (i.e., emergency room, ward, intensive care), the characteristics of the recovery rates in individuals after mild TBI should be investigated further.

The reported results underline the validity of the DSM-5 structure of PTSD as well as the comparability of PCL-5 scores across all tested language versions and different levels of recovery and severity of TBI. Hence, differences in test scores can be attributed to underlying ‘true’ differences in PTSD symptomatology rather than systematic sampling bias or measurement error. Future studies should examine the equivalence of PTSD assessments in additional subject groups and should investigate factors impacting PTSD symptomatology following TBI.

## Materials and methods

### Data

All the analyses in the present investigation utilized data from the Collaborative European NeuroTrauma Effectiveness Research in Traumatic Brain Injury (CENTER-TBI) project, supported by the European Union (EU) Framework 7 program (EC grant 602,150; clinicaltrials.gov NCT02210221)^38^. This prospective observational cohort study aimed to improve the characterization and clinical care of subjects after TBI. Data was sampled from the CENTER-TBI core study which comprises information on 4509 individuals who participated at 63 institutional sites across 18 countries between December 2014 and December 2017. The inclusion criteria for participation were a clinical diagnosis of TBI, indication for a computed tomography (CT) scan and presentation to the study center within 24 h post injury. Individuals with severe pre-existing neurological disorders (e.g., epilepsy, cerebrovascular accident) were excluded^52^.

The CENTER-TBI study was conducted in accordance with all relevant laws of the EU which were directly applicable or had a direct effect, as well as all the relevant laws of the countries in which the recruiting sites were located, including but not limited to, the relevant privacy and data protection laws and regulations (‘Privacy Law’), the relevant laws and regulations on the use of human materials, and all relevant guidelines relating to clinical studies including, but not limited to, the ICH Harmonized Tripartite Guideline for Good Clinical Practice (CPMP/ICH/135/95) (‘ICH GCP’) and the World Medical Association Declaration of Helsinki entitled ‘Ethical Principles for Medical Research Involving Human Subjects’. Ethical approval was attained for each recruitment site. Informed consent was obtained for all subjects recruited in the CENTER-TBI core study with documentation in electronic case report forms (e-CRF, QuesGen Systems Incorporated, Burlingame, CA, USA).

All methods employed in the current study were carried out in accordance with relevant guidelines and regulations. Furthermore, the experimental protocol of this study was approved by the management committee of CENTER-TBI: proposal #70, https://www.center-tbi.eu/data/approved-proposals.

### Ethical approval

The list of sites, ethics committees, approval numbers, and approval dates can be found on the official website of the CENTER-TBI project: www.center-tbi.eu/project/ethical-approval. The CENTER-TBI study received clearance from the following ethics committees: Ethikkommission der Medizinischen Universität Wien, Austria (1646/2014); Ethikkommission der Medizinischen Universität Innsbruck, Austria (AN2014-0,336,343/4.22); Centraal Ethisch Comité—Ethisch Comité Universitair Ziekenhuis Antwerpen en de Universiteit Antwerpen, Belgium (B300201422714); Comité d'Ethique Liège 412, Belgium (1427); Comité d'Ethique hospitalo-facultaire niversitaire de Liège 707, Belgium (B707201422102/2014–244); Comissie Medische Ethiek UZ KU Leuven, Belgium (B322201523981/S57019; ML11365); De Videnskabsetiske Komitéer for Region Syddanmark Odense/Copenhagen, Denmark (S-20140215); Varsinais suomen sairaanhoitopiirin kuntayhtyma—Eettinen Toimikunta Turku/Helsinki, Finland (95/1801/2014); Agence Nationale de Sécurité du Médicament et des Produits de Santé ANSM Paris/Besançon/Lille/Grenoble/Nancy/Poitiers, France (141421B-31); Ethikkommission Medizinsche Fakultät Heidelberg/Ludwigsburg, Germany (S-435/2014); Ethikkommission an der Medizinsche Fakultät Berlin, Germany (1098/15); Ethikkommission an der Medizinsche Fakultät Aachen, Germany (EK 174/15); ETT TUKEB Egészségügyi Tudományos Tanács Pecs/Szeged, Hungary (42,558–3/2014/EKU); Pécsi Tudományegyetem Pecs, Hungary (5421); Szegedi Tudományegyetem Szeged, Hungary (3803); Helsinki Committee, Rambam Health Care Campus Haifa, Israel (RMB 373-14); Hadassah Medical Organization IRB Jerusalem, Israel (0590-16 HMO); Fondazione IRCCS Ca' Granda Ospedale Maggiore Policlinico—Direzione Scientifica Comitato Etico Milan, Italy (542/2014); Comitato Etico—Ospedale San Raffaele Milan/Padova, Italy (217/2014); Comitato Etico Interaziendale A.O.U. Città della Salute e della Scienza di Torino—A.O. Ordine Mauriziano—A.S.L. Torino, Italy (0,015,269); Comitato Etico IRST IRCCS AVR Cesena, Italy (1675/2015 I.5/207); Comitato Etico Della Provincia Monza Brianza Monza, Italy (1978/2014); Comitato Etico Interaziendale A.O.U. ‘Maggiore della Carità’ Novara, Italy (CE 46/15); Comitato Etico—Ospedale Niguarda Ca’ Granda Milan, Italy (636–122,015); Ethics Commiitee for Clinical Research at Pauls Stradins Clinical University Hospital Development Society Riga/Rezekne, Latvia (171,215-1E); VILNIAUS REGIONINIS BIOMEDICININIŲ TYRIMŲ ETIKOS KOMITETAS Vilnius, Lithuania (158,200-15-801-323); KAUNO REGIONINIS BIOMEDICININIŲ TYRIMŲ ETIKOS KOMITETAS Kaunas, Lithuania (BE-2-6); Leids Universitair Centrum—Commissie Medische Ethiek Leiden/Rotterdam/the Hague/Nijmegen/Tilburg/Groningen, Netherlands (P14.222/NV/nv); Regional komité for medisinsk og helsefaglig Tromso/Trondheim/Oslo, Norway (2014/1454); Comitetului de Etica a Spitalului Clinic Judeteam de Urgenta Timisoara, Romania (16-OCT-2014); Etidkog odbora Klinidkog centra Vojvodine Novi Sad, Serbia (00-08/332); Comité Etico de Investigacion Clinica del Hospital Universitario 12 de Octubre Madrid, Spain (14/262); Comité ético de investigación clínica y comisión de proyectos de investigación del hospital universitari Vall d'Hebron Barcelona, Spain (ID-RTF080); Comité Etico de Investigacion Clinica de Euskadi Bilbao, Spain (PI2014158); Comité Etico de Investigacion Clinica del Clínico Universitario de Valencia, Spain (F-CE-GEva-15); EPN (Regionala Etikprövningsnämnden i Stockholm) Stockholm/Umea, Sweden (2014/1473-31/4); La Commission cantonale (VD) d’éthique de la recherche sur l'être humain (CER-VD) Lausanne, Switzerland (473/11); NHS HRA Birmingham/Cambridge/Southampton/Sheffield/London/Salford/Liverpool/Bristol, United Kingdom (14/SC/1370); UHB Research Governance Office—Queen Elizabeth Hospital Birmingham, United Kingdom (RRK5224); Research and Development Department—Cambridge University Hospital NHS Foundation Trust Cambridge, United Kingdom (AO93184); Research Governance Office—University Hospitals Southhampton NHS Trust Southampton, United Kingdom (RHM CRI0294); Research and Development Department—Sheffield Teaching Hospitals NHS Foundation Trust Sheffield, United Kingdom (STH18187); Research & Innovation Office—Kings college London NHS Foundation Trust London, United Kingdom (KCH15-204); Research and Development Department—Salford Royal Hospital NHS Foundation Trust Salford, United Kingdom (2015/025ET); Research & Innovation Office—The Walton centre NHS Foundation Trust Liverpool, United Kingdom (RG154-15); Research & Innovation—North Bristol NHS Trust Bristol, United Kingdom (3427); NHS Scotland Edinburgh, United Kingdom/Scotland (14/SS/1086); Research and Development Department—University Hospitals Division NHS Lothian Edinburgh, United Kingdom/Scotland (2015/0171).

### Study population

Data were extracted for adult subjects (age ≥ 16 years) who had completed psychopathological assessments at 6 months (− 1/ + 2 months) post injury. Subjects across the entire TBI severity spectrum were included in this study. Sociodemographic information was acquired at the time of enrollment into the CENTER-TBI study and included the subjects’ age, gender, marital status, education, occupation, self-reported pre-TBI history of psychiatric disorders, and cause of injury.

**Figure 1:**
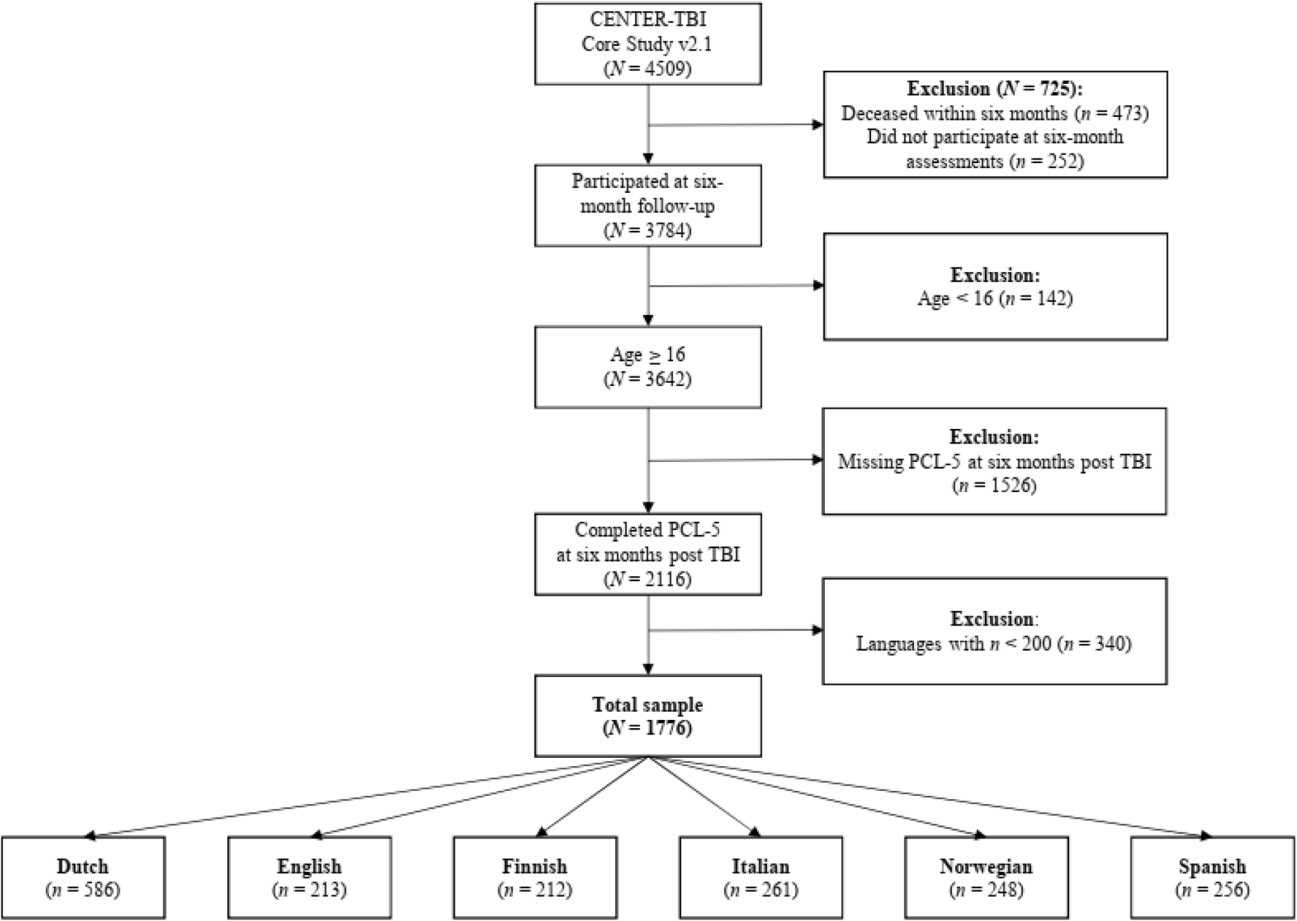
Sample attrition.

Participant data were aggregated by native language, further details can be found elsewhere^31^ and the application of MI analyses required the selection of language groups with a suitable sample size of n ≥ 200^40^. For details on the sample attrition in the current study, see Fig. 1.

### Injury-related variables

Extracranial injury severity was assessed using the Injury Severity Score (ISS) derived from the Abbreviated Injury Scale score^53,54^. ISS values can range from 0 to 75 with higher scores indicating greater impairment and the threshold for clinical impairment at 16. TBI severity was assessed at baseline by applying the Glasgow Coma Scale (GCS)^55^. GCS scores from 13 to 15 indicate mild, 9 to 12 moderate, and 3 to 6 severe TBI. Mild TBI can be further differentiated into complicated (GCS ≥ 13 with CT abnormalities) and uncomplicated (GCS ≥ 13 without CT abnormalities)^52^. Moreover, the Glasgow Outcome Scale Extended (GOSE)^56^ was administered as a clinician-reported measure of functional recovery at six months following TBI and was scored on an eight-point scale (1 = dead, 2 = vegetative state, 3/4 = lower/upper severe disability, 5/6 = lower/upper moderate disability, 7/8 = lower/upper good recovery). More details on GOSE data extraction are provided elsewhere^31^. Finally, in accordance with a recently reported procedure^51^ we considered individuals who had a complete recovery (GOSE = 8) from the mildest degree of TBI (GCS = 15) without any CT abnormalities as ‘ultra-mild’ cases in the TBI severity spectrum. This classification was used to determine whether the PCL-5 is able to capture PTSD symptomatology in the same way in those completely recovered compared to more severely injured subjects.

### PTSD symptoms

PTSD severity was evaluated using the PCL-5^20,23^. The PCL-5 is a self-report questionnaire comprising 20 PTSD symptoms which correspond to four diagnostic criteria proposed in the DSM-5 (i.e., criterion B: intrusion, criterion C: avoidance, criterion D: negative alterations in cognition and mood, criterion E: alterations in arousal and impulsivity)^12^. Individuals reported their impairment during the past month on a Likert scale ranging from 0 (not at all) to 4 (extremely). Total scores can range from 0 to 80 with higher values indicating greater symptom severity and can be used to screen for clinical levels of PTSD symptomatology with cutoffs of 31 to 33 in civilian populations^35,57^. In accordance with previous research in the field of TBI^45^, a screening cutoff of 31 was applied in the current study. Finally, to examine the nature of the traumatic experience (criterion A) associated with the PTSD symptoms, subjects were surveyed whether they completed the PCL-5 in reference to the TBI event (‘When you responded to the questions in this questionnaire were your answers in reference to the stressful experience which caused your traumatic brain injury?’).

The original version of the PCL-5 is openly available from the website of the National Center for PTSD: https://www.ptsd.va.gov/professional/assessment/adult-sr/ptsd-checklist.asp (last accessed on 17.11.2021). For the CENTER-TBI study the PCL-5 was translated and linguistically validated following a standardized protocol (for details, see^58^) as well as psychometrically tested^31^. All language versions can be retrieved from the CENTER-TBI website: https://www.center-tbi.eu/project/validated-translations-outcome-instruments (last accessed on 23.11.2021).

### Statistical analyses

Descriptive statistics are presented for sociodemographic characteristics as well as injury-related variables in the total sample and the language subsamples. Detailed information on the psychometric properties of the PCL-5 language versions in CENTER-TBI, both at the item level as well as at the scale level, can be found elsewhere^31^. Differences between language groups with respect to age were examined using an ANOVA and post-hoc Tukey HSD tests accounting for multiple comparisons. Differences in injury-related variables (i.e., GOSE, GCS, ISS, PCL-5) were tested via Kruskal–Wallis tests with post-hoc pairwise Mann–Whitney-U-tests and corrected for multiple comparisons (see Table A1). Effect sizes were determined by calculating Cohen’s d statistics^59^, whereby ds ≥ 0.2 indicate small, ds ≥ 0.5 moderate, and ds ≥ 0.8 large effects^60^.

The latent structure of the PCL-5 was investigated within the framework of confirmatory factor analyses (CFA) with robust weighted least square mean and variance (WLSMV) estimator for ordinal variables^61^. Fit analyses were conducted for the following candidate models: the original four-factor DSM-5 model of PTSD^12^, the four-factor Dysphoria model^27,46^, the six-factor Anhedonia model^23,62^, and the seven-factor Hybrid model^47,63,64^. Models were defined by mapping items to the respective proposed latent factors, including a common second-order factor to represent PTSD. Item mappings to the respective factors as proposed in these structural models are provided (Table B1 in Appendix). Standardized factors loadings were evaluated with a cutoff of β > 0.50. Model fit was evaluated based on multiple descriptive goodness-of-fit indices, namely the overall chi-square statistic, Comparative Fit Index (CFI), Tucker-Lewis Index (TLI), standardized root mean square residual (SRMR), and root mean square error of approximation (RMSEA) with a 90% confidence interval. Desirable fit was determined for CFI and TLI above 0.95, RMSEA below 0.06, and SRMR less than 0.08^65^. However, since these cutoff values were not originally proposed for WLSMV estimation of ordinal data, results should be interpreted cautiously^66^. We therefore evaluated structural validity for the candidate models considering all fit indices simultaneously.

The cross-linguistic equivalence of the PCL-5 assessments was investigated by applying multi-group CFA with a WLSMV estimator for ordinal data based on recommendations by Wu and Estabrook (2016)^67^ and adapted from Svetina, Rutkowski, and Rutkowski (2020)^68^. Three nested MI models were set up with increasingly constrained structural parameters: (1) configural model, (2) thresholds model, (3) loadings model. Models were defined by mapping items to the proposed latent factors and including between-factor covariances. Again, model fit was evaluated based on the previously described goodness-of-fit indices (i.e., chi-square, CFI, TLI, SRMR, RMSEA with 90% CI) in conjunction with the respective cutoffs. Likelihood ratio statistics of the relative model fit were examined using scaled chi-square difference tests with the Satorra-Bentler approximation^69^ and significance levels at α = 0.05. Significant differences would indicate rejection of the null hypothesis of equal model fit. However, since chi-square difference tests may overestimate effects in studies with large samples sizes^70^, changes in descriptive goodness-of-fit indices were evaluated as well. Based on previous recommendations^71,72^, between-model non-invariance was assumed for ΔCFI and ΔTLI ≥ 0.010, as well as ΔSRMR and ΔRMSEA ≥ 0.015. Final evaluations of the comparative model fit, and thus MI, were based on all relevant parameters concurrently.

Finally, MI analyses between individuals after ‘ultra-mild’ and more severe TBI were carried out in similar fashion as described above by employing increasingly constrained nested MI models and evaluated based on the same difference tests and model indices alongside the respective cutoffs.

The reported results are based on the ‘CENTER core 2.1’ dataset retrieved from the Neurobot platform of CENTER-TBI: https://center-tbi.incf.org (last accessed on 09.07.2021). All analyses were conducted in R 3.6.3^73^ using the packages ‘psych 2.0.12’^74^, ‘lavaan 0.6–8’^75^, and ‘semTools 0.5–4’^76^. For statistical tests, p < 0.05 was considered significant.

### Informed consent

Informed consent was obtained from all subjects involved in the study.

## Supplementary Information

Supplementary Information 1.

Supplementary Information 2.

Supplementary Information 3.

Supplementary Information 4.

Supplementary Information 5.

## Data Availability

All relevant data are available upon request from CENTER-TBI, and the authors are not legally allowed to share it publicly. The authors confirm that they received no special access privileges to the data. CENTER-TBI is committed to data sharing and in particular to responsible further use of the data. Hereto, we have a data sharing statement in place: https://www.center-tbi.eu/data/sharing. The CENTER-TBI Management Committee, in collaboration with the General Assembly, established the Data Sharing policy, and Publication and Authorship Guidelines to assure correct and appropriate use of the data as the dataset is hugely complex and requires help of experts from the Data Curation Team or Bio- Statistical Team for correct use. This means that we encourage researchers to contact the CENTER-TBI team for any research plans and the Data Curation Team for any help in appropriate use of the data, including sharing of scripts. Requests for data access can be submitted online: https://www.center-tbi.eu/data. The complete Manual for data access is also available online: https://www.center-tbi.eu/files/SOP-Manual-DAPR-20181101.pdf.

## Abbreviations

TBI: Traumatic brain injury
PTSD: Post-traumatic stress disorder
DSM-5: Diagnostic and statistical manual of mental disorders 5th edition
PCL-5: Post-traumatic stress disorder checklist for DSM-5
CFA: Confirmatory factor analysis
MI: Measurement invariance
CENTER-TBI: Collaborative European NeuroTrauma Effectiveness Research in TBI project
ISS: Injury severity score
GCS: Glasgow coma scale
GOSE: Glasgow outcome scale-extended
WLSMV: Weighted least square mean and variance estimator
CFI: Comparative fit index
TLI: Tucker-Lewis index
SRMR: Standardized root mean square residual
RMSEA: Root mean square error of approximation
RTA: Road traffic accidents
ER: Emergency room
ADM: Admission to ward
ICU: Intensive care unit
