# Supplementary material for: Measurement invariance of six language versions of the post-traumatic stress disorder checklist for DSM-5 in civilians after traumatic brain injury
Source: Sci Rep. 2022 Oct 4;12:16571. doi: 10.1038/s41598-022-20170-2 (PMC9532419; doi:10.1038/s41598-022-20170-2)
Supplement: Supplementary file 1 — Supplementary Information 1. [file 41598_2022_20170_MOESM1_ESM.docx]

**Appendix A – Mean difference tests of age and injury-related variables**

**Table A1.** Mean difference tests of age and injury-related variables across language subsamples

|  | Dutch | English | Finnish | Italian | Norwegian | Spanish |
| --- | --- | --- | --- | --- | --- | --- |
| Age: **F(5) = 6.86, p < .001, d = 0.36** | | | | | | |
| Dutch | - | **0.274** | **0.269** | 0.169 | 0.364 | 0.305 |
| English | **.013** | - | 0.005 | 0.093 | 0.104 | 0.042 |
| Finnish | **.011** | > .99 | - | 0.092 | 0.094 | 0.036 |
| Italian | .195 | .920 | .903 | - | 0.183 | 0.127 |
| Norwegian | < .001 | .892 | .911 | .255 | - | 0.059 |
| Spanish | < .001 | > .99 | > .99 | .660 | .985 | - |
| GCS: **H(5) = 38.24, p < .001, d = 0.28** | | | | | | |
| Dutch | - | **0.172** | 0.09 | **0.277** | 0.024 | 0.022 |
| English | **.004** | - | 0.317 | 0.057 | 0.246 | 0.161 |
| Finnish | > .99 | .063 | - | **0.285** | 0.053 | 0.013 |
| Italian | **< .001** | > .99 | **< .001** | - | **0.343** | 0.229 |
| Norwegian | > .99 | .102 | > .99 | **< .001** | - | 0.024 |
| Spanish | > .99 | > .99 | > .99 | .104 | > .99 | - |
| GOSE: **H(5) = 20.32, p = .001, d = 0.19** | | | | | | |
| Dutch | - | 0.153 | **0.219** | 0.052 | 0.007 | 0.013 |
| English | .273 | - | **0.390** | 0.112 | 0.212 | 0.156 |
| Finnish | **.018** | **.001** | - | **0.265** | 0.231 | 0.253 |
| Italian | > .99 | > .99 | **.036** | - | 0.072 | 0.034 |
| Norwegian | > .99 | .346 | .136 | > .99 | - | 0.028 |
| Spanish | > .99 | > .99 | .059 | > .99 | > .99 | - |
| ISS: **H(5) = 34.91, p < .001, d = 0.26** | | | | | | |
| Dutch | - | 0.123 | **0.320** | 0.201 | 0.065 | 0.044 |
| English | > .99 | - | **0.391** | 0.055 | 0.156 | 0.075 |
| Finnish | **< .001** | **.001** | - | **0.480** | 0.271 | **0.343** |
| Italian | .053 | > .99 | **< .001** | - | 0.228 | 0.149 |
| Norwegian | > .99 | > .99 | .056 | .155 | - | 0.083 |
| Spanish | > .99 | > .99 | **.004** | > .99 | > .99 | - |
| PCL-5 total: **H(5) = 28.05, p < .001, d = 0.23** | | | | | | |
| Dutch | - | 0.174 | 0.016 | **0.284** | 0.009 | **0.215** |
| English | .209 | - | 0.197 | 0.116 | 0.197 | 0.036 |
| Finnish | > .99 | .643 | - | **0.308** | 0.009 | 0.223 |
| Italian | **.001** | > .99 | **.014** | - | **0.311** | 0.076 |
| Norwegian | > .99 | .526 | > .99 | **.008** | - | 0.233 |
| Spanish | **.028** | > .99 | .245 | > .99 | .141 | - |

Note. GCS, Glasgow Coma Scale; GOSE, Glasgow Outcome Scale Extended; ISS, injury severity score; PCL-5, Posttraumatic Stress Disorder Checklist for DSM-5. Age differences across language subsamples were analyzed via ANOVA with post-hoc Tukey HSD tests. Differences in injury-related variables were analyzed via Kruskal-Wallis tests with post-hoc pairwise Mann-Whitney U tests and Bonferroni-adjusted p-values; p-values are shown in unfilled cells with corresponding Cohen’s d effect sizes in grey cells; bold values are significant at α = 0.05.
