# Supplementary material for: Measurement invariance of six language versions of the post-traumatic stress disorder checklist for DSM-5 in civilians after traumatic brain injury
Source: Sci Rep. 2022 Oct 4;12:16571. doi: 10.1038/s41598-022-20170-2 (PMC9532419; doi:10.1038/s41598-022-20170-2)
Supplement: Supplementary file 2 — Supplementary Information 2. [file 41598_2022_20170_MOESM2_ESM.docx]

**Appendix B – PCL-5 item mappings**

**Table B1.** PCL-5 item mappings for PTSD latent factor models (adapted from [52]).

| PCL-5 Item | DSM-5 model | Dysphoria model | Anhedonia model | Hybrid model |
| --- | --- | --- | --- | --- |
| (B1) Memories | IN | IN | IN | IN |
| (B2) Dreams | IN | IN | IN | IN |
| (B3) Flashbacks | IN | IN | IN | IN |
| (B4) Cued distress | IN | IN | IN | IN |
| (B5) Cued physical reactions | IN | IN | IN | IN |
| (C1) Avoiding internal reminders | AV | AV | AV | AV |
| (C2) Avoiding external reminders | AV | AV | AV | AV |
| (D1) Dissociative amnesia | NACM | D | NA | NA |
| (D2) Negative beliefs | NACM | D | NA | NA |
| (D3) Blame | NACM | D | NA | NA |
| (D4) Negative feelings | NACM | D | NA | NA |
| (D5) Loss of interest | NACM | D | ANH | ANH |
| (D6) Detachment or estrangement | NACM | D | ANH | ANH |
| (D7) Numbing | NACM | D | ANH | ANH |
| (E1) Irritability or aggressive behavior | AAR | D | DA | EB |
| (E2) Reckless behavior | AAR | D | DA | EB |
| (E3) Hypervigilance | AAR | AAR | AA | AA |
| (E4) Startle | AAR | AAR | AA | AA |
| (E5) Concentration | AAR | D | DA | DA |
| (E6) Sleep | AAR | D | DA | DA |

Note. AA, anxious arousal; AAR, alteration in arousal and reactivity (Cluster E); ANH, anhedonia; AV, avoidance (Cluster C); D, dysphoria; DA, dysphoric arousal; DSM-5, Diagnostic and Statistical Manual of Mental Disorders 5th edition; EB, externalizing behavior; IN = intrusion (Cluster B); NA, negative affect; NACM, negative alteration in cognition and mood (Cluster D); PCL-5, Posttraumatic Stress Disorder Checklist for DSM-5; PTSD, Posttraumatic Stress Disorder.
