# Supplementary material for: Measurement invariance of six language versions of the post-traumatic stress disorder checklist for DSM-5 in civilians after traumatic brain injury
Source: Sci Rep. 2022 Oct 4;12:16571. doi: 10.1038/s41598-022-20170-2 (PMC9532419; doi:10.1038/s41598-022-20170-2)
Supplement: Supplementary file 3 — Supplementary Information 3. [file 41598_2022_20170_MOESM3_ESM.docx]

**Appendix C – Multi-group CFA excluding the Finnish and Norwegian subsamples**

**Table C1.** Multi-group CFA results across language groups, excluding the Finnish and Norwegian subsamples (N = 1316).

| Model | χ^2^ | *df* | *p* | CFI | TLI | SRMR | RMSEA | RMSEA  90% CI | **Δ**CFI | **Δ**TLI | **Δ**SRMR | **Δ**RMSEA | **Δ**χ^2^ | **Δ**df | **Δ***p* |
| --- | --- | --- | --- | --- | --- | --- | --- | --- | --- | --- | --- | --- | --- | --- | --- |
| Configural | 1185.30 | 656 | <.001 | 0.996 | 0.995 | 0.059 | 0.050 | [0.045; 0.054] | **-** | **-** | **-** | **-** | **-** | **-** | **-** |
| Thresholds | 1262.50 | 776 | <.001 | 0.996 | 0.996 | 0.059 | 0.044 | [0.040; 0.048] | 0.000 | 0.001 | 0.000 | -0.006 | 97.98 | 120 | .93 |
| Loadings | 1308.57 | 824 | <.001 | 0.996 | 0.996 | 0.059 | 0.043 | [0.038; 0.047] | 0.000 | 0.000 | 0.000 | -0.001 | 24.17 | 48 | >.99 |

Note. Results are based on the original DSM-5 structure of PTSD [12]. Finnish and Norwegian subsamples were excluded from analyses due to a lack of cases with extreme impairment (response category 4) in at least one PCL-5 item. MI models are increasingly restricted and nested. The previous model always serves as a reference. CFA, confirmatory factor analysis; CFI, comparative fit index; ΔCFI, difference in CFI; CI, confidence interval; df, degrees of freedom; Δdf, difference in df; p, statistical significance of χ2; Δp, statistical significance of Δχ2; RMSEA, root mean square of approximation; ΔRMSEA, difference in RMSEA; SRMR, standard root mean square residual; ΔSRMR, difference in SRMR; TLI, Tucker-Lewis index; ΔTFI, difference in TFI; χ2, overall scaled chi-square statistic; Δχ2, scaled chi-square difference statistic.
