# Supplementary material for: Measurement invariance of six language versions of the post-traumatic stress disorder checklist for DSM-5 in civilians after traumatic brain injury
Source: Sci Rep. 2022 Oct 4;12:16571. doi: 10.1038/s41598-022-20170-2 (PMC9532419; doi:10.1038/s41598-022-20170-2)
Supplement: Supplementary file 4 — Supplementary Information 4. [file 41598_2022_20170_MOESM4_ESM.docx]

**Appendix D – Demographic and clinical characteristics in TBI severity groups**

**Table D1.** Demographic and clinical characteristics of individuals after ‘ultra-mild’ and more severe TBI [51].

|  |  | Ultra-Mild TBI | More Severe TBI |
| --- | --- | --- | --- |
| No. of cases | N (% of total) | 303 (17.06) | 1473 (82.94) |
| Age | M (SD) | 49.54 (19.64) | 49.42 (19.40) |
|  | Mdn (min; max) | 51 (16; 92) | 51 (16; 95) |
| Gender | female | 102 (33.66) | 519 (35.23) |
|  | male | 301 (66.34) | 954 (64.77) |
| Living situation | alone | 66 (21.78) | 301 (20.43) |
|  | not alone | 237 (78.22) | 1172 (79.57) |
| Education | none/primary | 36 (11.88) | 200 (13.58) |
|  | secondary | 79 (26.07) | 436 (29.60) |
|  | post-secondary | 171 (56.44) | 672 (45.62) |
|  | NA | 17 (5.61) | 165 (11.20) |
| Pre-TBI employment | full-time | 123 (40.60) | 620 (42.09) |
|  | part-time | 49 (16.17) | 161 (10.93) |
|  | in training | 30 (9.90) | 138 (9.37) |
|  | unemployed | 14 (4.62) | 115 (7.81) |
|  | retired | 77 (25.41) | 334 (22.67) |
|  | NA | 10 (3.30) | 105 (7.13) |
| Pre-TBI psychiatric history | yes | 32 (10.56) | 186 (12.63) |
|  | no | 271 (89.44) | 1274 (86.49) |
|  | NA | 0 (0.00) | 13 (0.88) |
| TBI cause | incidental fall | 157 (51.82) | 616 (41.82) |
|  | RTA | 101 (33.33) | 644 (43.72) |
|  | other | 43 (14.19) | 184 (12.49) |
|  | NA | 2 (0.66) | 29 (1.97) |
| Clinical care pathways | ER | 183 (60.40) | 226 (15.34) |
|  | ADM | 107 (35.31) | 585 (39.71) |
|  | ICU | 13 (4.29) | 662 (44.94) |
| Loss of consciousness | yes | 151 (49.84) | 893 (60.62) |
|  | no | 131 (43.23) | 436 (29.60) |
|  | NA | 21 (6.93) | 144 (9.78) |
| TBI severity | uncomplicated mild | 303 (100) | 311 (21.11) |
|  | complicated mild | 0 (0.00) | 536 (36.39) |
|  | moderate | 0 (0.00) | 127 (8.62) |
|  | severe | 0 (0.00) | 262 (17.79) |
|  | NA | 0 (0.00) | 237 (16.09) |
| GCS at baseline | M (SD) | 15.00 (0.00) | 12.50 (3.92) |
|  | Mdn (min; max) | 15 (15; 15) | 15 (3; 15) |
| Recovery at six months (GOSE) | good recovery | 303 (100) | 856 (58.11) |
|  | moderate disability | 0 (0.00) | 457 (31.03) |
|  | severe disability | 0 (0.00) | 159 (10.79) |
|  | NA | 0 (0.00) | 1 (0.07) |
| Extracranial  Injury Severity Score (ISS) | M (SD) | 7.39 (6.20) | 20.38 (15.04) |
|  | Mdn (min; max) | 5 (1; 34) | 16 (1; 75) |
| PCL-5 referring to TBI event | yes | 176 (58.09) | 992 (67.35) |
|  | no | 114 (37.62) | 435 (29.53) |
|  | NA | 13 (4.29) | 46 (3.12) |
| PTSD Symptoms (PCL-5) | M (SD) | 6.73 (9.95) | 13.23 (14.15) |
|  | Mdn (min; max) | 3 (0; 50) | 8 (0; 72) |
| Provisional PTSD (PCL-5 ≥ 31) | yes | 16 (5.28) | 174 (11.81) |
|  | no | 287 (94.72) | 1299 (88.19) |

*Note*. For continuous variables and total scores, mean (M) with standard deviation (SD) and median (Mdn) with range (min; max) are reported; ADM, admission to ward; ER, emergency room; GCS, Glasgow Coma Scale; GOSE, Glasgow Outcome Scale Extended; ICU, intensive care unit; ISS, injury severity score; NA, not available; PCL-5, total score on Posttraumatic Stress Disorder Checklist for DSM-5; PTSD, Posttraumatic Stress Disorder; RTA, road traffic accident; TBI, traumatic brain injury.
