# Supplementary material for: Measurement invariance of six language versions of the post-traumatic stress disorder checklist for DSM-5 in civilians after traumatic brain injury
Source: Sci Rep. 2022 Oct 4;12:16571. doi: 10.1038/s41598-022-20170-2 (PMC9532419; doi:10.1038/s41598-022-20170-2)
Supplement: Supplementary file 5 — Supplementary Information 5. [file 41598_2022_20170_MOESM5_ESM.docx]

**The CENTER-TBI participants and investigators:**

Cecilia Åkerlund^7^, Krisztina Amrein^8^, Nada Andelic^9^, Lasse Andreassen^10^, Audny Anke^11^, Anna Antoni^12^, Gérard Audibert^13^, Philippe Azouvi^14^, Maria Luisa Azzolini^15^, Ronald Bartels^16^, Pál Barzó^17^, Romuald Beauvais^18^, Ronny Beer^19^, Bo-Michael Bellander^20^, Antonio Belli^21^, Habib Benali^22^, Maurizio Berardino^23^, Luigi Beretta^15^, Morten Blaabjerg^24^, Peter Bragge^25^, Alexandra Brazinova^26^, Vibeke Brinck^27^, Joanne Brooker^28^, Camilla Brorsson^29^, Andras Buki^30^, Monika Bullinger^31^, Manuel Cabeleira^32^, Alessio Caccioppola^33^, Emiliana Calappi ^33^, Maria Rosa Calvi^15^, Peter Cameron^34^, Guillermo Carbayo Lozano^35^, Marco Carbonara^33^, Simona Cavallo^23^, Giorgio Chevallard^36^, Arturo Chieregato^36^, Giuseppe Citerio^37, 38^, Hans Clusmann^39^, Mark Coburn^40^, Jonathan Coles^41^, Jamie D. Cooper^42^, Marta Correia^43^, Amra Čović ^1^, Nicola Curry^44^, Endre Czeiter^30^, Marek Czosnyka^32^, Claire Dahyot‑Fizelier^45^, Paul Dark^46^, Helen Dawes^47^, Véronique De Keyser^48^, Vincent Degos^22^, Francesco Della Corte^49^, Hugo den Boogert^16^, Bart Depreitere^50^, Đula Đilvesi ^51^, Abhishek Dixit^52^, Emma Donoghue^28^, Jens Dreier^53^, Guy‑Loup  Dulière^54^, Ari Ercole^52^, Patrick Esser^47^, Erzsébet Ezer^55^, Martin  Fabricius^56^, Valery L. Feigin^57^, Kelly  Foks^58^, Shirin Frisvold^59^, Alex Furmanov^60^, Pablo Gagliardo^61^, Damien Galanaud^22^, Dashiell Gantner^34^, Guoyi Gao^62^, Pradeep George^63^, Alexandre Ghuysen^64^, Lelde Giga^65^, Ben Glocker^66^, Jagoš Golubovic^51^, Pedro A. Gomez ^67^, Johannes Gratz^68^, Benjamin Gravesteijn^69^, Francesca Grossi^49^, Russell L. Gruen^70^, Deepak Gupta^71^, Juanita A. Haagsma^69^, Iain Haitsma^72^, Raimund Helbok^19^, Eirik Helseth^73^, Lindsay Horton ^74^, Jilske Huijben^69^, Peter J. Hutchinson^75^, Bram Jacobs^76^, Stefan Jankowski^77^, Mike Jarrett^27^, Ji‑yao  Jiang^63^, Faye Johnson^78^, Kelly Jones^57^, Mladen Karan^51^, Angelos G. Kolias^75^, Erwin Kompanje^79^, Daniel Kondziella^56^, Evgenios Kornaropoulos^52^, Lars‑Owe Koskinen^80^, Noémi Kovács^81^, Ana Kowark^82^, Alfonso Lagares^67^, Linda Lanyon^63^, Steven Laureys^83^, Fiona Lecky^84, 85^, Didier Ledoux^83^, Rolf Lefering^86^, Valerie Legrand^87^, Aurelie Lejeune^88^, Leon Levi^89^, Roger Lightfoot^90^, Hester Lingsma^69^, Andrew I.R. Maas^48^, Ana M. Castaño‑León^67^, Marc Maegele^91^, Marek Majdan^26^, Alex Manara^92^, Geoffrey Manley^93^, Costanza Martino^94^, Hugues Maréchal^54^, Julia Mattern^95^, Catherine McMahon^96^, Béla Melegh^97^, David Menon^52^, Tomas Menovsky^48^, Ana Mikolic^69^, Benoit Misset^83^, Visakh Muraleedharan^63^, Lynnette Murray^34^, Ancuta Negru^98^, David Nelson^7^, Virginia Newcombe^52^, Daan Nieboer^69^, József Nyirádi^8^, Otesile Olubukola^84^, Matej Oresic^99^, Fabrizio Ortolano^33^, Aarno Palotie^100, 101, 102^, Paul M. Parizel^103^, Jean‑François Payen^104^, Natascha Perera^18^, Vincent Perlbarg^22^, Paolo Persona^105^, Wilco Peul^106^, Anna Piippo-Karjalainen^107^, Matti Pirinen^100^, Dana Pisica^69^, Horia Ples^98^, Suzanne Polinder^69^, Inigo Pomposo^35^, Jussi P. Posti ^108^, Louis Puybasset^109^, Andreea Radoi ^110^, Arminas Ragauskas^111^, Rahul Raj^107^, Malinka Rambadagalla^112^, Isabel Retel Helmrich^69^, Jonathan Rhodes^113^, Sylvia Richardson^114^, Sophie Richter^52^, Samuli Ripatti^100^, Saulius Rocka^111^, Cecilie Roe^115^, Olav Roise^116,117^, Jonathan Rosand^118^, Jeffrey V. Rosenfeld^119^, Christina Rosenlund^120^, Guy Rosenthal^60^, Rolf Rossaint^82^, Sandra Rossi^105^, Daniel Rueckert^66^ Martin Rusnák^121^, Juan Sahuquillo^110^, Oliver Sakowitz^95, 122^, Renan Sanchez‑Porras^122^, Janos Sandor^123^, Nadine Schäfer^86^, Silke Schmidt^124^, Herbert Schoechl^125^, Guus Schoonman^126^, Rico Frederik Schou^127^, Elisabeth Schwendenwein^12^, Charlie Sewalt^69^, Ranjit D. Singh^106^, Toril Skandsen^128, 129^ , Peter Smielewski^32^, Abayomi Sorinola^130^, Emmanuel Stamatakis^52^, Simon Stanworth^44^, Robert Stevens^131^, William Stewart^132^, Ewout W. Steyerberg^69,^ ^133^, Nino Stocchetti^134^, Nina Sundström^135^, Riikka Takala^136^, Viktória Tamás^130^, Tomas Tamosuitis^137^, Mark Steven Taylor^26^, Braden Te Ao^57^, Olli Tenovuo^108^, Alice Theadom^57^, Matt Thomas^92^, Dick Tibboel^138^, Marjolein Timmers^79^, Christos Tolias^139^, Tony Trapani^34^, Cristina Maria Tudora^98^, Andreas Unterberg^95^, Peter Vajkoczy ^140^, Shirley Vallance^34^, Egils Valeinis^65^, Zoltán Vámos^55^, Mathieu van der Jagt^141^, Gregory Van der Steen^48^, Joukje van der Naalt^76^, Jeroen T.J.M. van Dijck ^106^, Inge A. M. van Erp^106^, Thomas A. van Essen^106^, Wim Van Hecke^142^, Caroline van Heugten^143^, Dominique Van Praag^2^, Ernest van Veen^69^, Thijs Vande Vyvere^142^, Roel P. J. van Wijk^106^, Alessia Vargiolu^38^, Emmanuel Vega^88^, Kimberley Velt^69^, Jan Verheyden^142^, Paul M. Vespa^144^, Anne Vik^128, 145^, Rimantas Vilcinis^137^, Victor Volovici^72^, Nicole von Steinbüchel^1^, Daphne Voormolen^69^, Petar Vulekovic^51^, Kevin K.W. Wang^146^, Daniel Whitehouse^52^, Eveline Wiegers^69^, Guy Williams^52^, Lindsay Wilson^74^, Stefan Winzeck^52^, Stefan Wolf^147^, Zhihui Yang^118^, Peter Ylén^148^, Alexander Younsi^95^, Frederick A. Zeiler^52,149^, Veronika Zelinkova^26^, Agate Ziverte^65^ , Tommaso Zoerle^33^

Numbering continued on from the original manuscript

1. Department of Physiology and Pharmacology, Section of Perioperative Medicine and Intensive Care, Karolinska Institutet, Stockholm, Sweden
2. János Szentágothai Research Centre, University of Pécs, Pécs, Hungary
3. Division of Surgery and Clinical Neuroscience, Department of Physical Medicine and Rehabilitation, Oslo University Hospital and University of Oslo, Oslo, Norway
4. Department of Neurosurgery, University Hospital Northern Norway, Tromso, Norway
5. Department of Physical Medicine and Rehabilitation, University Hospital Northern Norway, Tromso, Norway
6. Trauma Surgery, Medical University Vienna, Vienna, Austria
7. Department of Anesthesiology & Intensive Care, University Hospital Nancy, Nancy, France
8. Raymond Poincare hospital, Assistance Publique – Hopitaux de Paris, Paris, France
9. Department of Anesthesiology & Intensive Care, S Raffaele University Hospital, Milan, Italy
10. Department of Neurosurgery, Radboud University Medical Center, Nijmegen, The Netherlands
11. Department of Neurosurgery, University of Szeged, Szeged, Hungary
12. International Projects Management, ARTTIC, Munchen, Germany
13. Department of Neurology, Neurological Intensive Care Unit, Medical University of Innsbruck, Innsbruck, Austria
14. Department of Neurosurgery & Anesthesia & intensive care medicine, Karolinska University Hospital, Stockholm, Sweden
15. NIHR Surgical Reconstruction and Microbiology Research Centre, Birmingham, UK
16. Anesthesie-Réanimation, Assistance Publique – Hopitaux de Paris, Paris, France
17. Department of Anesthesia & ICU, AOU Città della Salute e della Scienza di Torino - Orthopedic and Trauma Center, Torino, Italy
18. Department of Neurology, Odense University Hospital, Odense, Denmark
19. BehaviourWorks Australia, Monash Sustainability Institute, Monash University, Victoria, Australia
20. Department of Public Health, Faculty of Health Sciences and Social Work, Trnava University, Trnava, Slovakia
21. Quesgen Systems Inc., Burlingame, California, USA
22. Australian & New Zealand Intensive Care Research Centre, Department of Epidemiology and Preventive Medicine, School of Public Healthand Preventive Medicine, Monash University, Melbourne, Australia
23. Department of Surgery and Perioperative Science, Umeå University, Umeå, Sweden
24. Department of Neurosurgery, Medical School, University of Pécs, Hungary and Neurotrauma Research Group, János Szentágothai ResearchCentre, University of Pécs, Hungary
25. Department of Medical Psychology, Universitätsklinikum Hamburg-Eppendorf, Hamburg, Germany
26. Brain Physics Lab, Division of Neurosurgery, Dept of Clinical Neurosciences, University of Cambridge, Addenbrooke’s Hospital, Cambridge, UK
27. Neuro ICU, Fondazione IRCCS Cà Granda Ospedale Maggiore Policlinico, Milan, Italy
28. ANZIC Research Centre, Monash University, Department of Epidemiology and Preventive Medicine, Melbourne, Victoria, Australia
29. Department of Neurosurgery, Hospital of Cruces, Bilbao, Spain
30. NeuroIntensive Care, Niguarda Hospital, Milan, Italy
31. School of Medicine and Surgery, Università Milano Bicocca, Milano, Italy
32. NeuroIntensive Care, ASST di Monza, Monza, Italy
33. Department of Neurosurgery, Medical Faculty RWTH Aachen University, Aachen, Germany
34. Department of Anesthesiology and Intensive Care Medicine, University Hospital Bonn, Bonn, Germany
35. Department of Anesthesia & Neurointensive Care, Cambridge University Hospital NHS Foundation Trust, Cambridge, UK
36. School of Public Health & PM, Monash University and The Alfred Hospital, Melbourne, Victoria, Australia
37. Radiology/MRI department, MRC Cognition and Brain Sciences Unit, Cambridge, UK
38. Oxford University Hospitals NHS Trust, Oxford, UK
39. Intensive Care Unit, CHU Poitiers, Potiers, France
40. University of Manchester NIHR Biomedical Research Centre, Critical Care Directorate,  Salford Royal Hospital NHS Foundation Trust, Salford, UK
41. Movement Science Group, Faculty of Health and Life Sciences, Oxford Brookes University, Oxford, UK
42. Department of Neurosurgery, Antwerp University Hospital and University of Antwerp, Edegem, Belgium
43. Department of Anesthesia & Intensive Care, Maggiore Della Carità Hospital, Novara, Italy
44. Department of Neurosurgery, University Hospitals Leuven, Leuven, Belgium
45. Department of Neurosurgery, Clinical centre of Vojvodina, Faculty of Medicine, University of Novi Sad, Novi Sad, Serbia
46. Division of Anaesthesia, University of Cambridge, Addenbrooke’s Hospital, Cambridge, UK
47. Center for Stroke Research Berlin, Charité – Universitätsmedizin Berlin, corporate member of Freie Universität Berlin, Humboldt-Universität zu Berlin, and Berlin Institute of Health, Berlin, Germany
48. Intensive Care Unit, CHR Citadelle, Liège, Belgium
49. Department of Anaesthesiology and Intensive Therapy, University of Pécs, Pécs, Hungary
50. Departments of Neurology, Clinical Neurophysiology and Neuroanesthesiology, Region Hovedstaden Rigshospitalet, Copenhagen, Denmark
51. National Institute for Stroke and Applied Neurosciences, Faculty of Health and Environmental Studies, Auckland University of Technology, Auckland, New Zealand
52. Department of Neurology, Erasmus MC, Rotterdam, the Netherlands
53. Department of Anesthesiology and Intensive care, University Hospital Northern Norway, Tromso, Norway
54. Department of Neurosurgery, Hadassah-hebrew University Medical center, Jerusalem, Israel
55. Fundación Instituto Valenciano de Neurorrehabilitación (FIVAN), Valencia, Spain
56. Department of Neurosurgery, Shanghai Renji hospital, Shanghai Jiaotong University/school of medicine, Shanghai, China
57. Karolinska Institutet, INCF International Neuroinformatics Coordinating Facility, Stockholm, Sweden
58. Emergency Department, CHU, Liège, Belgium
59. Neurosurgery clinic, Pauls Stradins Clinical University Hospital, Riga, Latvia
60. Department of Computing, Imperial College London, London, UK
61. Department of Neurosurgery, Hospital Universitario 12 de Octubre, Madrid, Spain
62. Department of Anesthesia, Critical Care and Pain Medicine, Medical University of Vienna, Austria
63. Department of Public Health, Erasmus Medical Center-University Medical Center, Rotterdam, The Netherlands
64. College of Health and Medicine, Australian National University, Canberra, Australia
65. Department of Neurosurgery, Neurosciences Centre & JPN Apex trauma centre, All India Institute of Medical Sciences, New Delhi-110029, India
66. Department of Neurosurgery, Erasmus MC, Rotterdam, the Netherlands
67. Department of Neurosurgery, Oslo University Hospital, Oslo, Norway
68. Division of Psychology, University of Stirling, Stirling, UK
69. Division of Neurosurgery, Department of Clinical Neurosciences, Addenbrooke’s Hospital & University of Cambridge, Cambridge, UK
70. Department of Neurology, University of Groningen, University Medical Center Groningen, Groningen, Netherlands
71. Neurointensive Care , Sheffield Teaching Hospitals NHS Foundation Trust, Sheffield, UK
72. Salford Royal Hospital NHS Foundation Trust Acute Research Delivery Team, Salford, UK
73. Department of Intensive Care and Department of Ethics and Philosophy of Medicine, Erasmus Medical Center, Rotterdam, The Netherlands
74. Department of Clinical Neuroscience, Neurosurgery, Umeå University, Umeå, Sweden
75. Hungarian Brain Research Program - Grant No. KTIA_13_NAP-A-II/8, University of Pécs, Pécs, Hungary
76. Department of Anaesthesiology, University Hospital of Aachen, Aachen, Germany
77. Cyclotron Research Center , University of Liège, Liège, Belgium
78. Centre for Urgent and Emergency Care Research (CURE), Health Services Research Section, School of Health and Related Research (ScHARR), University of Sheffield, Sheffield, UK
79. Emergency Department, Salford Royal Hospital, Salford UK
80. Institute of Research in Operative Medicine (IFOM), Witten/Herdecke University, Cologne, Germany
81. VP Global Project Management CNS, ICON, Paris, France
82. Department of Anesthesiology-Intensive Care, Lille University Hospital, Lille, France
83. Department of Neurosurgery, Rambam Medical Center, Haifa, Israel
84. Department of Anesthesiology & Intensive Care, University Hospitals Southhampton NHS Trust, Southhampton, UK
85. Cologne-Merheim Medical Center (CMMC), Department of Traumatology, Orthopedic Surgery and Sportmedicine, Witten/Herdecke University,Cologne, Germany
86. Intensive Care Unit, Southmead Hospital, Bristol, Bristol, UK
87. Department of Neurological Surgery, University of California, San Francisco, California, USA
88. Department of Anesthesia & Intensive Care,M. Bufalini Hospital, Cesena, Italy
89. Department of Neurosurgery, University Hospital Heidelberg, Heidelberg, Germany
90. Department of Neurosurgery, The Walton centre NHS Foundation Trust, Liverpool, UK
91. Department of Medical Genetics, University of Pécs, Pécs, Hungary
92. Department of Neurosurgery, Emergency County Hospital Timisoara , Timisoara, Romania
93. School of Medical Sciences, Örebro University, Örebro, Sweden
94. Institute for Molecular Medicine Finland, University of Helsinki, Helsinki, Finland
95. Analytic and Translational Genetics Unit, Department of Medicine; Psychiatric & Neurodevelopmental Genetics Unit, Department of Psychiatry; Department of Neurology, Massachusetts General Hospital, Boston, MA, USA
96. Program in Medical and Population Genetics; The Stanley Center for Psychiatric Research, The Broad Institute of MIT and Harvard, Cambridge, MA, USA
97. Department of Radiology, University of Antwerp, Edegem, Belgium
98. Department of Anesthesiology & Intensive Care, University Hospital of Grenoble, Grenoble, France
99. Department of Anesthesia & Intensive Care, Azienda Ospedaliera Università di Padova, Padova, Italy
100. Dept. of Neurosurgery, Leiden University Medical Center, Leiden, The Netherlands and Dept. of Neurosurgery, Medical Center Haaglanden, The Hague, The Netherlands
101. Department of Neurosurgery, Helsinki University Central Hospital
102. Division of Clinical Neurosciences, Department of Neurosurgery and Turku Brain Injury Centre, Turku University Hospital and University of Turku, Turku, Finland
103. Department of Anesthesiology and Critical Care, Pitié -Salpêtrière Teaching Hospital, Assistance Publique, Hôpitaux de Parisand University Pierre et Marie Curie, Paris, France
104. Neurotraumatology and Neurosurgery Research Unit (UNINN), Vall d'Hebron Research Institute, Barcelona, Spain
105. Department of Neurosurgery, Kaunas University of technology and Vilnius University, Vilnius, Lithuania
106. Department of Neurosurgery, Rezekne Hospital, Latvia
107. Department of Anaesthesia, Critical Care & Pain Medicine NHS Lothian & University of Edinburg, Edinburgh, UK
108. Director, MRC Biostatistics Unit, Cambridge Institute of Public Health, Cambridge, UK
109. Department of Physical Medicine and Rehabilitation, Oslo University Hospital/University of Oslo, Oslo, Norway
110. Division of Orthopedics, Oslo University Hospital, Oslo, Norway
111. Institue of Clinical Medicine, Faculty of Medicine, University of Oslo, Oslo, Norway
112. Broad Institute, Cambridge MA Harvard Medical School, Boston MA, Massachusetts General Hospital, Boston MA, USA
113. National Trauma Research Institute, The Alfred Hospital, Monash University, Melbourne, Victoria, Australia
114. Department of Neurosurgery, Odense University Hospital, Odense, Denmark
115. International Neurotrauma Research Organisation, Vienna, Austria
116. Klinik für Neurochirurgie, Klinikum Ludwigsburg, Ludwigsburg, Germany
117. Division of Biostatistics and Epidemiology, Department of Preventive Medicine, University of Debrecen, Debrecen, Hungary
118. Department Health and Prevention, University Greifswald, Greifswald, Germany
119. Department of Anaesthesiology and Intensive Care, AUVA Trauma Hospital, Salzburg, Austria
120. Department of Neurology, Elisabeth-TweeSteden Ziekenhuis, Tilburg, the Netherlands
121. Department of Neuroanesthesia and Neurointensive Care, Odense University Hospital, Odense, Denmark
122. Department of Neuromedicine and Movement Science, Norwegian University of Science and Technology, NTNU, Trondheim, Norway
123. Department of Physical Medicine and Rehabilitation, St.Olavs Hospital, Trondheim University Hospital, Trondheim, Norway
124. Department of Neurosurgery, University of Pécs, Pécs, Hungary
125. Division of Neuroscience Critical Care, John Hopkins University School of Medicine, Baltimore, USA
126. Department of Neuropathology, Queen Elizabeth University Hospital and University of Glasgow, Glasgow, UK
127. Dept. of Department of Biomedical Data Sciences, Leiden University Medical Center, Leiden, The Netherlands
128. Department of Pathophysiology and Transplantation, Milan University, and Neuroscience ICU, Fondazione IRCCS Cà Granda OspedaleMaggiore Policlinico, Milano, Italy
129. Department of Radiation Sciences, Biomedical Engineering, Umeå University, Umeå, Sweden
130. Perioperative Services, Intensive Care Medicine and Pain Management, Turku University Hospital and University of Turku, Turku,Finland
131. Department of Neurosurgery, Kaunas University of Health Sciences, Kaunas, Lithuania
132. Intensive Care and Department of Pediatric Surgery, Erasmus Medical Center, Sophia Children’s Hospital, Rotterdam, The Netherlands
133. Department of Neurosurgery, Kings college London, London, UK
134. Neurologie, Neurochirurgie und Psychiatrie, Charité – Universitätsmedizin Berlin, Berlin, Germany
135. Department of Intensive Care Adults, Erasmus MC– University Medical Center Rotterdam, Rotterdam, the Netherlands
136. icoMetrix NV, Leuven, Belgium
137. Movement Science Group, Faculty of Health and Life Sciences, Oxford Brookes University, Oxford, UK
138. Director of Neurocritical Care, University of California, Los Angeles, USA
139. Department of Neurosurgery, St.Olavs Hospital, Trondheim University Hospital, Trondheim, Norway
140. Department of Emergency Medicine, University of Florida, Gainesville, Florida, USA
141. Department of Neurosurgery, Charité – Universitätsmedizin Berlin, corporate member of Freie Universität Berlin, Humboldt-Universitätzu Berlin, and Berlin Institute of Health, Berlin, Germany
142. VTT Technical Research Centre, Tampere, Finland
143. Section of Neurosurgery, Department of Surgery, Rady Faculty of Health Sciences, University of Manitoba, Winnipeg, MB, Canada
